# Arabidopsis Transcriptome Analysis Reveals Key Roles of Melatonin in Plant Defense Systems

**DOI:** 10.1371/journal.pone.0093462

**Published:** 2014-03-28

**Authors:** Sarah Weeda, Na Zhang, Xiaolei Zhao, Grace Ndip, Yangdong Guo, Gregory A. Buck, Conggui Fu, Shuxin Ren

**Affiliations:** 1 Agriculture Research Station, Virginia State University, Petersburg, Virginia, United States of America; 2 College of Agriculture and Biotechnology, China Agricultural University, Beijing, P. R. China; 3 Tianjin Crop Science Institute, Tianjin Academy of Agricultural Science, Tianjin, P. R. China; 4 Department of Chemistry, Virginia State University, Petersburg, Virginia, United States of America; 5 Center of the Study of Biological Complexity, Virginia Commonwealth University, Richmond, Virginia, United States of America; University of North Carolina at Charlotte, United States of America

## Abstract

Melatonin is a ubiquitous molecule and exists across kingdoms including plant species. Studies on melatonin in plants have mainly focused on its physiological influence on growth and development, and on its biosynthesis. Much less attention has been drawn to its affect on genome-wide gene expression. To comprehensively investigate the role(s) of melatonin at the genomics level, we utilized mRNA-seq technology to analyze Arabidopsis plants subjected to a 16-hour 100 pM (low) and 1 mM (high) melatonin treatment. The expression profiles were analyzed to identify differentially expressed genes. 100 pM melatonin treatment significantly affected the expression of only 81 genes with 51 down-regulated and 30 up-regulated. However, 1 mM melatonin significantly altered 1308 genes with 566 up-regulated and 742 down-regulated. Not all genes altered by low melatonin were affected by high melatonin, indicating different roles of melatonin in regulation of plant growth and development under low and high concentrations. Furthermore, a large number of genes altered by melatonin were involved in plant stress defense. Transcript levels for many stress receptors, kinases, and stress-associated calcium signals were up-regulated. The majority of transcription factors identified were also involved in plant stress defense. Additionally, most identified genes in ABA, ET, SA and JA pathways were up-regulated, while genes pertaining to auxin responses and signaling, peroxidases, and those associated with cell wall synthesis and modifications were mostly down-regulated. Our results indicate critical roles of melatonin in plant defense against various environmental stresses, and provide a framework for functional analysis of genes in melatonin-mediated signaling pathways.

## Introduction

Melatonin (*N*-acetyl-5-methoxytryptamine) is a versatile molecule that, since its 1958 discovery in bovine pineal glands [1], has been found across kingdom lines including bacteria, fungi, and plants [2]–[5]. Its numerous and diverse functions include regulating circadian rhythms and seasonal reproduction cycles, promoting sleep, preventing diabetes, attenuating cancer cell proliferation, and enhancing immunity [6]–[10]. Melatonin has also been characterized as a potent antioxidant capable of scavenging reactive oxygen species (ROS) and reactive nitrogen species (RNS) [11]–[14].

Since the relatively recent discovery of melatonin in plants, investigations to elucidate its function in plants has been driven by what is known in animals. One such area of focus is the involvement of melatonin in modulating circadian rhythms and photoperiod-dependent processes. While melatonin levels do appear to be affected by light/dark cycles in some plants, the pattern varies among species, tissues, and organs [15]–[21]. The nocturnal peak of melatonin characteristic of humans and animals has been observed in *Chenopodium rubrum* [16], grape skins [17] and *Ulva* sp [21]. Conversely, studies conducted in water hyacinth demonstrated a peak in melatonin levels late in the day [19], indicating its biosynthesis in light. Furthermore, melatonin biosynthesis occurred under constant light in senescent rice leaves and was nearly undetectable under constant darkness [22]. Other reports found no significant correlation with melatonin levels and day/night cycles [18]. Interestingly, developing sweet cherries exhibited a dual peak of melatonin levels, one nocturnal and one in late day [20]. Contradicting reports of melatonin levels in ripening fruits add to the variation observed among plant species; melatonin levels decreased in ripening cherries [20], but increased in ripening tomatoes [18]. The possible role of melatonin in regulating flowering has also been investigated [23]–[25]; however an unequivocal role of melatonin in photoperiod-dependent processes in plants has not yet been established

Melatonin has been studied extensively as an antioxidant in mammals. Many studies demonstrate the ability of melatonin to protect against many human diseases, including those linked to oxidative stress [26]–[27]. Melatonin was able to attenuate paraquat-induced lung and liver damage in rats [28]–[29] and Parkinson's disease in mice [30]. Furthermore, exogenously applied melatonin can enhance the production of antioxidative enzymes such as glutathione peroxidase and superoxide dismutase [31]. Melatonin may similarly play a protective role against oxidative stress in plants. Oxidative stress is capable of inducing elevated melatonin levels in various plant species [17], [32]–[34]. Indeed, the daytime peak of melatonin levels found in sweet cherry was associated with high temperature and light intensity, suggesting melatonin was synthesized in response to oxidative stress [17]. Transgenic rice seedlings with elevated levels of melatonin were more resistant to herbicide induced oxidative stress than their wild type counterparts [35]. Furthermore, oxidative stress induced the expression of genes involved in melatonin biosynthesis, leading to increased melatonin production in both wild type and transgenic rice [35]. Melatonin also appears to protect plants against UV and ozone damage [36]–[40], attenuate photo-oxidation of the photosynthetic system, and, at moderate levels, protect chlorophyll during senescence [39]–[42]. In addition, melatonin can promote low temperature and osmotic stress tolerance [43]–[48], alleviate copper damage [49]–[50], and improve salt tolerance [51] and fungal disease resistance [52] in a diversity of plant species.

The structure of melatonin is another feature that has driven investigations into its function in plants. Melatonin is structurally similar to the plant hormone indole-3-acetic acid (IAA) and has many features that make it a candidate for a functional auxin [53]–[54]. In addition, melatonin and auxin biosynthetic pathways share the same precursor, tryptophan [55]. Since auxins play critical roles as growth regulators during plant development such as shoot elongation, lateral root formation, and cell expansion, much work has focused on the effect of melatonin on these processes [42], [48], [56]–[63]. Investigations have shown that melatonin and its precursor serotonin affect root growth in a dose-dependent manner similar to auxin [56]–[60]. At low melatonin levels, lateral root growth is stimulated, while at higher levels, adventitious root formation occurs and lateral root growth is inhibited in a mechanism seemingly independent of auxin [64]. Furthermore, melatonin has been demonstrated to stimulate expansion of etiolated lupin cotyledons [62] and promote hypocotyl growth [61], [63] similar to IAA. It is still unknown whether the auxin-like affects are due to the action of melatonin itself or if melatonin is converted into IAA [64]. Moreover, while mammalian systems have well documented receptor-mediated gene expression, melatonin receptors have not been identified in plants and evidence points to a chemical response rather than a receptor-dependent response [64].

While much of the work conducted on melatonin in plants has focused on its physiological influence on growth and development, and on its biosynthesis, little work has focused on its affect on gene expression. Microarray analysis using endogenous melatonin-rich transgenic rice identified several hundred genes that are up- or down- regulated by elevated melatonin levels [65]. Previously, in an effort to understand mechanisms on how melatonin promotes lateral root formation in cucumber, we conducted mRNA-seq analysis using cucumber root tissues and identified potential clusters of genes that may control melatonin-mediated lateral root formation [66]. In this study, using Arabidopsis as a model, we utilized next generation RNA sequencing technology (RNA-seq) to obtain a comprehensive analysis of genome-wide changes in responses to external application of melatonin. RNA-seq can detect changes in gene expression with more precision than a standard microarray, allowing for the potential to identify novel genes. As a model species, Arabidopsis has many advantages for both basic and applied research, including easy transformation and ample resources of available T-DNA lines. Systemic analysis of the effect melatonin has on genome-wide gene expression in Arabidopsis will provide us basic information to genetically dissect melatonin-mediated signaling pathway(s) in regulating plant growth and development.

## Materials and Methods

### Plant materials and growth condition

*Arabidopsis thaliana* ecotype Columbia (Col-0) was used for RNA-seq experiments and oxidative stress experiments. All plants were grown in a growth-chamber under 23°C and 14/10 hours light/dark cycle. Three-week-old seedlings were removed from soil, rinsed, and submerged in100 pM, or 1 mM melatonin for 16 hours with gentle shaking. Mock solution was used as control. All experiments were duplicated for statistical analysis.

### RNA extraction, library construction and sequencing

After melatonin treatment, total RNA was extracted using the QIAGEN RNeasy Mini Kit according to the manufacturer's instructions (QIAGEN). Purification of mRNA from total RNA was conducted using an Oligotex mRNA Mini Kit (QIAGEN). The mRNA was then used to construct cDNA libraries using the mRNA-Seq Sample Preparation Kit™ (Illumina) following standard protocols. Briefly, the mRNA was fragmented by exposure to divalent cations at 94°C and the fragmented mRNA was converted into double stranded cDNA. The cDNA ends were polished, the 3′-hydroxls extended with A bases, and ligated to Illumina-specific adapter-primers. The adaptor ligated DNA was amplified by 15 cycles of PCR followed by purification using Qiagen™ PCR purification kit to obtain the final library for sequencing. The DNA yield and fragment insert size distribution of the library were determined on the Agilent Bioanalyzer. Library quantifications were performed by qPCR assays using the KAPA Library Quant Kit™ following the manufacturer's instructions.

All six constructed libraries were loaded on the Illumina flow cell at the appropriate concentration and bridge amplified to create millions of individual clonal clusters. The flow cells were sequenced on the HiSeq2000 sequencing instrument using 50 b single end protocols at the Center for the Study of Biological Complexity of the Virginia Commonwealth University.

### Post-sequence analysis

After high throughput sequencing performance, short prematurely terminated sequences and those with low quality, as well as reads with any ambiguous bases were removed from raw sequence reads. Clean reads were aligned to the Arabidopsis genome assembly TAIR10 (as a reference) using TopHat (v1.3.1) BWA software [67]. Alignment results were stored in SAM/BAM file format [68] to permit downstream analysis. Cufflinks (v1.0.3) software [69] was then applied to identify transcripts and their abundance levels in RNA-seq data, as well as to perform differential gene expression analysis among multiple samples. Cufflinks calculates expression levels in fragments per kilobase of sequence per million fragments mapped (FPKM), which were used to perform differential gene expression analysis among treatments [69].

Gene Ontology classification of all genes exhibiting significant changes in transcript levels was conducted using AmiGO online tool [70]. Those with only CUFF numbers assigned during automatic analysis were manually checked against their positions using TAIR resources. Gene IDs and their putative functions were assigned accordingly.

### Validation of mRNA-seq data using qRT-PCR

A total of 60 genes were selected for verification of mRNA-seq data using qRT-PCR. Primer pairs for each selected gene were presented in Table S1. One microgram RNase free DNase treated total RNA from seedlings treated or untreated with melatonin was used to synthesize first strand cDNA using Superscript III (Invitrogen). qRT-PCR was performed using SSoAdvanced SYBR Green Supermix (BioRad) with the following parameters: 95°C for 3 minutes followed by 40 cycles of 95°C for 10 seconds and 60°C for 30 seconds. Gene expression was normalized via the Livak method using Arabidopsis Elongation Factor 1 (EF1; AT5G60390) as a reference gene [71].

### Paraquat-induced oxidative stress

The ability of melatonin to attenuate paraquat-induced oxidative stress was examined using detached Arabidopsis leaves. Seedlings were grown under short day conditions (10/14 hour light/dark photoperiod) to promote vegetative growth for four weeks. Leaves were then detached and incubated in 0 mM, 10 mM or 50 mM paraquat in the presence or absence of 1 mM melatonin under 16/8 hour light/dark photoperiod. After 48 hours, leaves were analyzed for oxidative stress as visualized by photobleaching.

## Results and Discussion

### Transcriptome sequencing and identification of up- and down-regulated genes by melatonin

To elucidate the roles of melatonin in regulating genome-wide gene expression, we conducted mRNA-seq analysis using Arabidopsis after 16 hours melatonin treatment. Because melatonin may function as a hormone at low concentration, and an antioxidant at high concentrations [35], [37], [56], we chose 100 pM (low) and 1 mM (high) melatonin to treat Arabidopsis to capture what may be physiological levels in *planta* and elevated levels. Mock solution was used as control. Biological duplicates for each treatment and control were included. After removing short prematurely terminated sequences, low quality sequences and any ambiguous bases, a total of more than 10 million clean reads with at least 10× transcriptome coverage for each library (controls, 100 pM melatonin and 1 mM melatonin treatments) were generated through RNA-seq sequencing (Table 1). Of these, 87.1% and 88.3% for control, 88.0% and 88.6% for 100 pM treatment, and 87.0% and 87.9% for 1 mM treatment were mapped to the Arabidopsis genome assembly TARI10 using TopHat software [67]. Cufflinks was then used to assemble the aligned reads into transcripts and estimate the abundance of the transcripts to analyze differential expression among treatments [69]. In 100 pM melatonin-treated seedlings, only 81 gene transcript levels changed significantly (p<0.05). Of those genes, 30 were up-regulated, 51 were down-regulated. In addition, 4 genes were completely suppressed and 5 genes were uniquely expressed compared to controls. Of the genes that exhibited a change in transcript levels at least 2-fold, 26 were up-regulated and 38 were down-regulated. However, when treated with 1 mM melatonin, more genes were identified with altered expression. A total of 1308 genes exhibited significant changes (p<0.05) in transcript levels in response to 1 mM melatonin with 566 genes up-regulated, and 742 genes down-regulated. Five genes were identified that were uniquely expressed in melatonin treated seedlings while 11 were completely suppressed by 1 mM melatonin. Nearly 900 genes exhibited changes in gene expression of at least 2-fold in response to 1 mM melatonin (387 up-regulated, 510 down-regulated). The lists of these genes are presented in additional files as Table S2 for low melatonin affected genes and Table S3 for high melatonin affected genes. All these genes with significant changes in expression levels in response to low or high concentrations of melatonin were also identified using TAIR database and classified according to their functions using the Gene Ontology GO slimmer.

**Table 1:** Total number of clean and mapped reads and bases for each sample following mRNA-seq.

SAMPLE	CLEAN READS	MAPPED READS	% MAPPED	TOTAL BASES	MAPPED BASES	TRANSCRIPTOME COVERAGE
Control 1	16777955	14617162	87.1	855675705	745475262	14.3×
100 pM Mel 1	17302219	15224369	88.0	882413169	776442819	14.7×
1 mM Mel 1	12395079	10782224	87.0	632149029	549893424	10.5×
Control 2	12383964	10938665	88.3	631582164	557871915	10.5×
100 pM Mel 2	18378365	16281804	88.6	937296615	830372004	15.6×
1 mM Mel 2	17452335	15331176	87.9	890069085	781889976	14.8×

Sequences were mapped to the Arabidopsis TAIR10 genome using TopHat.

### Reproducibility of RNA-seq profiles

**Figure 1:**
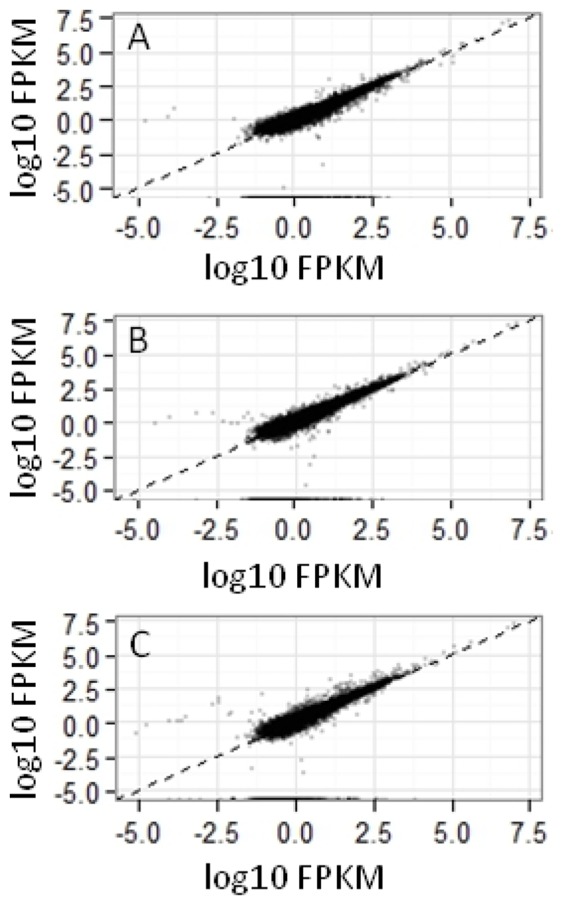
Scatter plots show genomic scale reproducibility. The scatter plots comparing the clean reads of two biological duplicates from control (A), 100 pM melatonin (B) and 1 mM melatonin (C) treatments. Genes are represented by dots. For each gene, the RNA expression level in one rep is given on the x axis and the same gene in the other rep is given on the y axis.

To examine the variability among our RNA-seq experiments, all clean reads from melatonin treated and control sets were plotted for all possible pairs of independent experiments. Scatter plots of these data for control plants, 100 pM and 1 mM melatonin-treated plants are shown in Figure 1 and Figure S1. These scatter plots show that the most genes exhibit less variation in expression between the biological duplicates (Figure 1) when compared to the scatter plots between treatments (Figure S1). To further confirm its reproducibility, a phylogenetic analysis using sequences from all 6 libraries was conducted. Again, the biologically duplicated treatments are more closely related than those of the different treatments (Figure S2). Taken together, these results indicate that our experiments are highly reproducible.

### Validation of sequencing data by qRT-PCR

**Figure 2:**
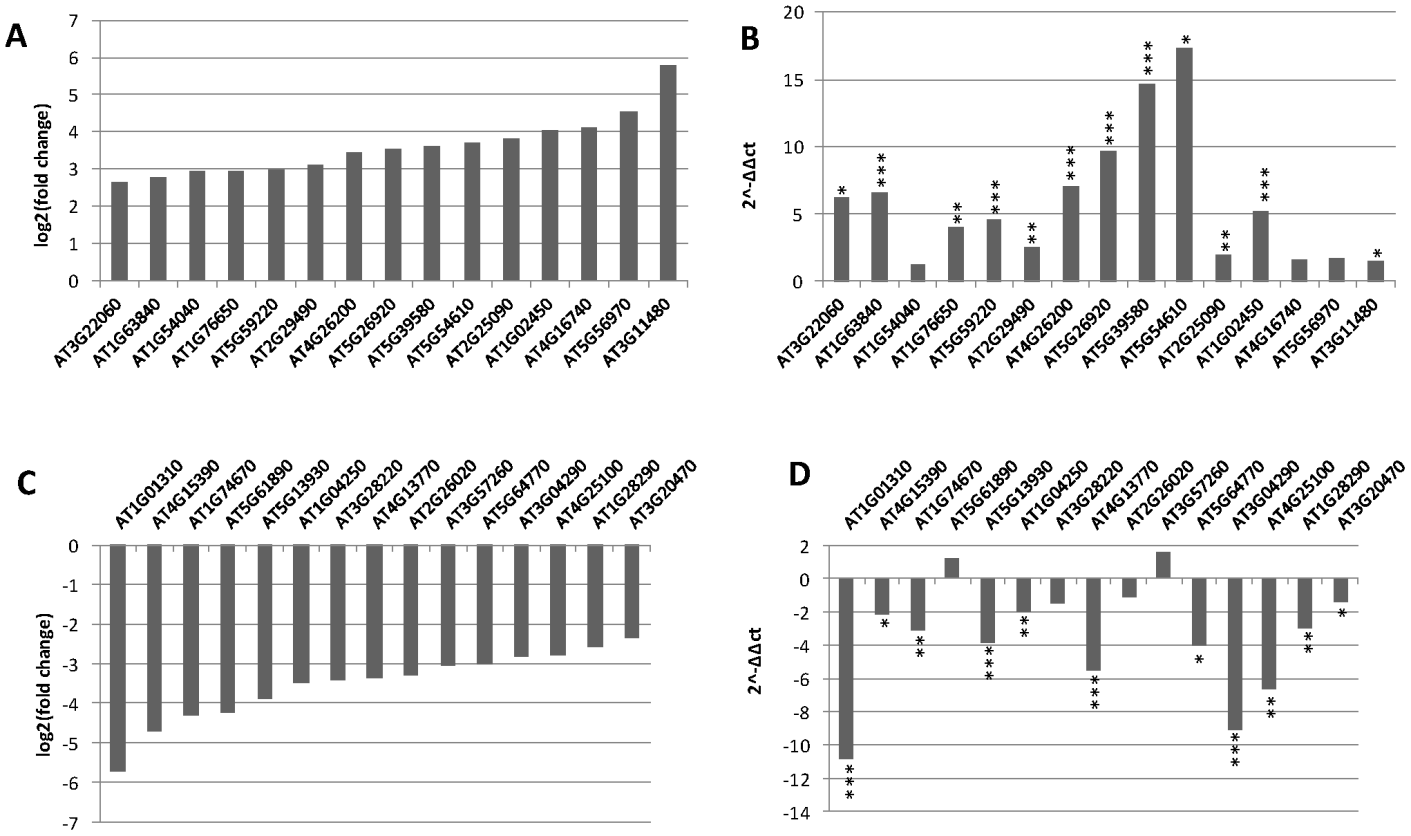
qRT-PCR analysis confirming overall results of RNA-seq experiments. The fold changes in transcript levels identified in RNA-seq experiments for 15 selected genes are graphed in A (up-regulated) and C (down-regulated). qRT-PCR was performed using the same samples for RNA-seq experiments with primers for the selected genes showing up-regulated (B) or down-regulated (D) by 1 mM melatonin. All q-RT-PCR were repeated four times. * p<0.05, ** p<0.01, ***p<0.001.

Although the results indicated that our experiments are highly reproducible, we further examined the reliability of the observed changes between treatments. qRT-PCR experiments were performed for a total of 60 genes exhibiting expression changes in response to melatonin treatments in the mRNA-seq analysis. The transcript levels were measured using the same RNAs used for the RNA-seq transcriptome analysis. The full list of 60 genes and their qRT-PCR validation is included in Table S4. Comparisons between mRNA-seq data and qRT-PCR for the top 15 up- and down-regulated genes from the selected group are shown in Figure 2. As shown, most of the selected genes indicated as differentially expressed by RNA-seq transcriptome profiling were confirmed by qRT-PCR. Only 7 of the selected genes were found to not significantly change in response to 1 mM melatonin, although the mRNA-seq data found significant changes in their transcript levels. However, relative fold changes observed in mRNA-seq data are sometimes not comparable to qRT-PCR analysis. This is mainly due to limitations and sensitivity of RNA-seq analysis and global normalization methods. Nevertheless, the qRT-PCR analysis fit well with the mRNA-seq data, indicating the reliability of the RNA-seq data.

### Annotation and classification of differentially expressed genes into functional categories

The Gene Ontology (GO) classification was conducted for all genes exhibiting a significant change in transcript levels using the AmiGO Slimmer tool and TAIR database. The Slimmer tool assigned general parent (GO slim) terms to the genes according to biological process, cellular component, and molecular function. The most striking difference between up and down regulated genes are those involved in response to stress and responses to endogenous and biotic stimuli. Of the up-regulated genes that were classified to a GO slim term, approximately 42% were involved in response to stress, 25% in response to endogenous stimulus, 24% in response to biotic stimulus, and 22% in response to signal transduction (Table 2). In down-regulated genes, only 17% were involved in response to stress, 9% in response to endogenous stimulus, and 7% in response to both biotic stimulus and signal transduction. Interestingly, this trend was not observed in response to 100 pM melatonin. The proportion of genes involved in response to stress and biotic stimulus following 100 pM melatonin treatment was approximately 30% and 14%, respectively, for both up- and down-regulated genes. The trend was reversed in cell communication and signal transduction as a greater proportion of genes involved in those processes were down regulated in response to 100 pM melatonin. Genes involved in photosynthesis also trended towards down-regulation in response to 1 mM melatonin but trended towards up-regulation in response to 100 pM. Furthermore, genes associated with the cell wall accounted for 7% of down-regulated genes but only 3% of up-regulated genes. 9% of genes down-regulated and 6% of the up-regulated genes with changes of at least 2-fold were associated with redox pathways.

**Table 2:** Gene ontology classification into biological process of all genes significantly (p<0.05) differentially expressed in response to 1 mM and 100 pM melatonin.

		1 mM Melatonin		100 pM Melatonin	
GO Slim ID	Biological Process	up-regulated	down-regulated	up-regulated	down-regulated
GO:0009987	cellular process	57.73	57.40	43.33	55.32
GO:0008152	metabolic process	50.72	51.59	50.00	53.19
GO:0006950	response to stress	42.27	16.74	30.00	29.79
GO:0009058	biosynthetic process	28.60	35.82	23.33	31.91
GO:0009719	response to endogenous stimulus	25.00	9.13	23.33	12.77
GO:0009607	response to biotic stimulus	23.92	6.64	13.33	14.89
GO:0007154	cell communication	23.56	8.85	6.67	14.89
GO:0006810	transport	23.20	14.38	10.00	12.77
GO:0007165	signal transduction	21.94	6.50	3.33	10.64
GO:0009628	response to abiotic stimulus	21.76	17.43	23.33	17.02
GO:0008150	Biological process *	18.88	23.79	23.33	21.28
GO:0016043	cellular component organization	15.65	17.15	3.33	17.02
GO:0019538	protein metabolic process	12.59	11.62	3.33	19.15
GO:0009056	catabolic process	12.05	10.79	20.00	6.38
GO:0007275	multicellular organismal development	11.87	17.29	10.00	12.77
GO:0008219	cell death	10.61	1.94	3.33	8.51
GO:0019748	secondary metabolic process	8.99	7.61	10.00	4.26
GO:0006139	nucleobase-containing compound metabolic process	8.45	22.13	13.33	14.89
GO:0006464	cellular protein modification process	7.37	7.05	0	10.64
GO:0009605	response to external stimulus	6.83	4.01	3.33	6.38
GO:0006629	lipid metabolic process	5.58	9.68	13.33	8.51
GO:0000003	reproduction	4.86	5.39	0	4.26
GO:0009791	post-embryonic development	4.86	9.68	3.33	4.26
GO:0005975	carbohydrate metabolic process	4.32	13.69	3.33	4.26
GO:0009653	anatomical structure morphogenesis	4.14	10.93	3.33	4.26
GO:0009991	response to extracellular stimulus	3.60	1.94	3.33	6.38
GO:0040007	growth	3.60	5.81	0	4.26
GO:0016049	cell growth	2.70	3.46	0	2.13
GO:0030154	cell differentiation	2.34	5.39	3.33	2.13
GO:0019725	cellular homeostasis	1.98	2.49	3.33	0
GO:0006091	generation of precursor metabolites and energy	1.62	8.16	3.33	0
GO:0009908	flower development	1.62	3.04	0.00	4.26
GO:0009790	embryo development	1.26	1.52	0.00	2.13
GO:0015979	photosynthesis	1.26	9.68	6.67	2.13
GO:0007049	cell cycle	1.08	1.80	0	2.13
GO:0006259	DNA metabolic process	0.90	2.21	0	0
GO:0009838	abscission	0.54	0.14	0	2.13
GO:0009856	pollination	0.54	0.41	0	0
GO:0040029	regulation of gene expression, epigenetic	0.36	1.11	0	2.13
GO:0006412	translation	0.18	1.80	0	4.26
GO:0009606	tropism	0.18	0.69	0	0
GO:0007267	cell-cell signaling	0	0.28	0	0
GO:0009875	pollen-pistil interaction	0	0.14	0	0

Genes were classified using AmiGO GO slimmer. The proportion of genes assigned to each category was calculated by dividing the number of genes assigned to a category by the total number of differentially expressed genes for each treatment. Genes may be classified into one or more GO Slim Term.

*Indicates biological process is unknown.

The biological processes that trended towards down-regulation in response to 1 mM melatonin included biosynthetic processes, metabolism of carbohydrates and nucleobase-containing compounds, development, cellular organization, morphogenesis, photosynthesis, and generation of precursor metabolites and energy.

The cellular components associated with genes down-regulated in response to 1 mM melatonin included cytoplasm, extracellular region, and, consistent with the down-regulation of photosynthesis associated genes, the thylakoid and plastids (Table 3). The cellular components assigned to genes that trended towards up-regulation in response to 1 mM melatonin were the nucleus, plasma membrane, and the Golgi apparatus. The molecular functions associated with genes up-regulated in response to 1 mM melatonin included transferase activity, protein binding, and kinase activity, consistent with the general trend of signaling (Table 4). The molecular functions that trended towards down-regulation in response to 1 mM melatonin were hydrolase activity, nucleic acid (both DNA and RNA) binding, lipid binding, and structural molecule activity.

**Table 3:** Gene ontology classification into cellular component of all genes significantly (p<0.05) differentially expressed in response to 1 mM and 100 pM Melatonin.

		1 mM Melatonin		100 pM Melatonin	
GO Slim ID	Cellular Component	up-regulated	down regulated	up-regulated	down regulated
GO:0005623	cell	80.04	77.04	80.00	63.83
GO:0005622	intracellular	66.19	67.77	73.33	53.19
GO:0005737	cytoplasm	44.78	51.87	60.00	34.04
GO:0005634	nucleus	27.16	20.89	16.67	17.02
GO:0016020	membrane	25.36	26.83	20.00	12.77
GO:0005886	plasma membrane	20.32	11.34	3.33	10.64
GO:0005576	extracellular region	13.67	20.19	10.00	23.40
GO:0009536	plastid	12.77	30.71	43.33	17.02
GO:0005739	mitochondrion	10.07	8.44	6.67	6.38
GO:0005575	Cellular component *	8.63	9.54	13.33	2.13
GO:0005829	cytosol	6.65	5.26	3.33	4.26
GO:0005794	Golgi apparatus	6.12	2.07	3.33	4.26
GO:0005618	cell wall	5.94	5.39	3.33	6.38
GO:0030312	external encapsulating structure	5.94	5.39	3.33	6.38
GO:0005773	vacuole	5.04	4.56	20.00	4.26
GO:0005783	endoplasmic reticulum	2.52	2.21	3.33	0
GO:0005768	endosome	0.72	0.55	0	0
GO:0005730	nucleolus	0.54	0.69	3.33	2.13
GO:0005777	peroxisome	0.54	0.83	0	0
GO:0009579	thylakoid	0.54	11.48	10.00	0
GO:0005654	nucleoplasm	0.36	0.00	0	2.13
GO:0005635	nuclear envelope	0.18	0.28	0	0
GO:0005615	extracellular space	0	0.14	0	0
GO:0005764	lysosome	0	0	0	0
GO:0005840	ribosome	0	1.38	3.33	0
GO:0005856	cytoskeleton	0	0.41	0.00	0

The proportion of genes in each category were calculated by dividing the number of genes assigned to a category by the total number of genes that were classified in a treatment by the AmiGO GO slimmer.

*Indicates cellular component is unknown.

**Table 4:** Gene Ontology classification into molecular function of all genes significantly (p<0.05) differentially expressed in response to 1 mM and 100 pM melatonin.

		1 mM Melatonin		100 pM Melatonin	
GO Slim ID	Molecular Function	up-regulated	down regulated	up-regulated	down regulated
GO:0003824	catalytic activity	37.41	32.23	43.33	46.81
GO:0005488	binding	25.72	21.85	23.33	25.53
GO:0003674	Molecular function *	21.04	24.76	30.00	21.28
GO:0016740	transferase activity	16.73	9.82	13.33	21.28
GO:0016787	hydrolase activity	12.05	10.93	23.33	10.64
GO:0005515	protein binding	11.33	7.05	13.33	8.51
GO:0016301	kinase activity	7.73	3.18	0	6.38
GO:0003700	sequence-specific DNA binding transcription factor activity	6.83	5.39	0	2.13
GO:0005215	transporter activity	5.94	4.56	3.33	2.13
GO:0003676	nucleic acid binding	4.32	7.33	0	8.51
GO:0000166	nucleotide binding	3.96	2.21	0	8.51
GO:0003677	DNA binding	3.24	5.39	0	4.26
GO:0008289	lipid binding	1.44	1.80	0	0
GO:0019825	oxygen binding	1.26	0.97	0	2.13
GO:0030234	enzyme regulator activity	1.26	1.24	3.33	4.26
GO:0004871	signal transducer activity	0.72	1.11	0	0
GO:0030246	carbohydrate binding	0.72	0.55	0	0
GO:0004872	receptor activity	0.54	0	0	0
GO:0003723	RNA binding	0.36	1.52	0	2.13
GO:0004518	nuclease activity	0.36	0.41	3.33	0
GO:0005102	receptor binding	0.36	0.14	0	0
GO:0003682	chromatin binding	0	0	0	0
GO:0003774	motor activity	0	0.83	0	0
GO:0005198	structural molecule activity	0	1.80	0	0
GO:0008135	translation factor activity, nucleic acid binding	0	0.14	0	0

Genes were classified into at least one category using AmiGO GO slimmer. The proportion of genes that fall into each category were calculated by dividing the number of genes assigned to a category by the total number of genes that could be classified for each treatment.

*Indicates Molecular function is unknown.

Interestingly, expression of chlorophyllase (CLH1), a light regulated enzyme involved in chlorophyll degradation [72], was significantly down-regulated in response to 1 mM melatonin (Gene ID: AT1G19670 in Table S3). This is consistent with a study conducted on senescing apple leaves where exogenous melatonin inhibited transcript levels of pheide a oxygnease (PAO), another key enzyme involved in chlorophyll degradation [41]. These findings can thus explain the means by which melatonin preserves chlorophyll content in leaves [39]–[42], delays senescence [39]–[41], and enhances photosynthetic rates [41]. Indeed, this may provide another mechanism by which melatonin preserves chlorophyll content during senescence in addition to attenuating ROS.

Due to the classic function of melatonin as a “clock” hormone in animals, one might infer that it would play a central role in timing of events in plants, such as flowering. However, since less than 1% of the genes affected by melatonin were involved in flowering, but nearly 40% were involved in stress responses and signaling, it is tempting to deduce that the central role of melatonin in plants is more likely as an antioxidant involved in stress response and as a photo-protectant. However, since the melatonin treatment was a one-time event and flowering is a complex programmed event that is promoted over several days, we cannot completely rule out that fluctuations in melatonin levels over time may signal day length and promote flowering.

Much fewer genes were affected by low level (100 pM) of melatonin treatment with only 81 genes significantly altered their expression. In view of the biological processes, most of the identified genes involved in cellular process, cell communication, response to stress, transporters and signal transductions are down-regulated. Cell death associated genes identified were also mostly down-regulated by 100 pM melatonin.

**Figure 3:**
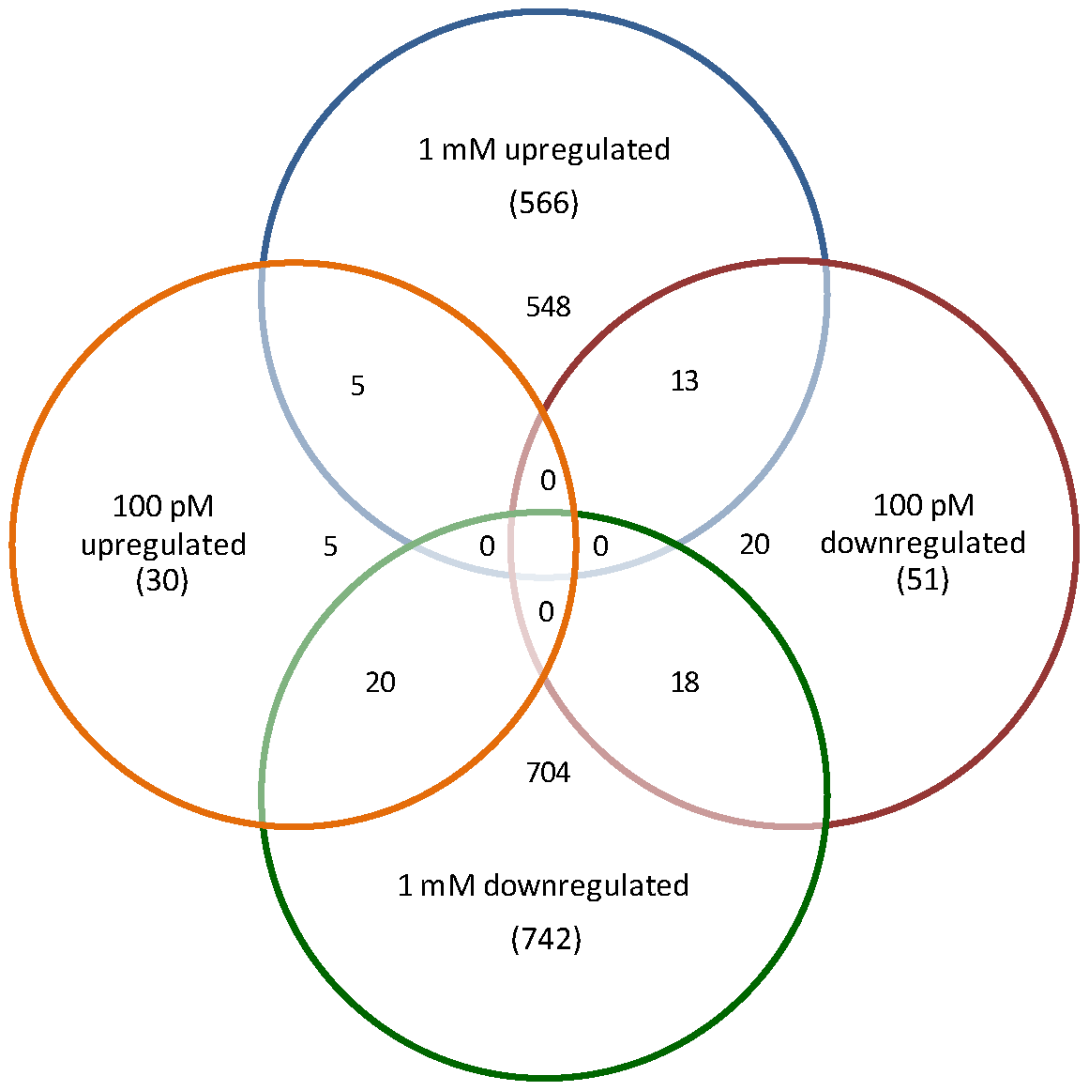
Venn Diagram representing the overlap of the molecular responses to low (100 pM) and high (1 mM) melatonin treatments. The number in the parenthesis indicates the number of genes altered by melatonin for each treatment.

Comparative analysis was conducted on genes affected by 100 pM melatonin and those by 1 mM melatonin. Results showed that not all genes down-regulated by low (100 pM) melatonin were down regulated by high (1 mM) melatonin. In fact, out of 51 of 100 pM down-regulated genes, only 18 genes were down regulated by 1 mM melatonin treatment, and 13 genes were up-regulated by 1 mM melatonin treatment. Similarly, out of 30 genes up-regulated by 100 pM melatonin, only 5 were also up-regulated by 1 mM melatonin and 8 were down regulated by 1 mM melatonin. Seventeen genes were uniquely up-regulated and 20 were uniquely down-regulated by low (100 pM) melatonin treatment (Figure 3). These results suggest that melatonin may play significantly different roles in control of plant growth and development under low and high concentrations. Overall, the results indicate that melatonin plays important physiological roles in many biological processes during plant growth and development such as stress defense, photosynthesis, cell wall modification and redox homeostasis.

### Identification of novel transcriptionally active regions affected by melatonin

One of the advantages of mRNA-seq technique over microarray is that it can discover novel genes that were not previously annotated [73], [74]. By mapping RNA-seq reads (contigs) against TAIR, we identified 13 contigs that were located in regions of the Arabidopsis genome where there were not predicted genes. Most of these contigs very strong FPKM signals, indicating they are legitimate transcripts. Three genes at positions 2:11563932–11571856, 1:13838585–13839136, and 2:12241769–12242729 were up-regulated by melatonin. Seven at positions 4:2716534–2717374, 5:10330615–10331733, 5:14550372–14550814, 5:18701853–18702020, 5:18891010–18891182, 5:19624545–19624871, and 5:22455105–22455271 were significantly down regulated by melatonin. In addition, 3 regions at positions 11670600–11670738 and 11672189–11672349 on chromosome 1, and 12241769–12242729 on chromosome 2 were completely suppressed by 1 mM melatonin treatment. Further detailed studies are requisite to determine whether these transcriptionally active regions are protein-encoding genes or noncoding RNAs, and to elucidate their roles in regulating melatonin-mediated plant physiological responses.

### Melatonin altered many genes involved in plant defense

**Figure 4:**
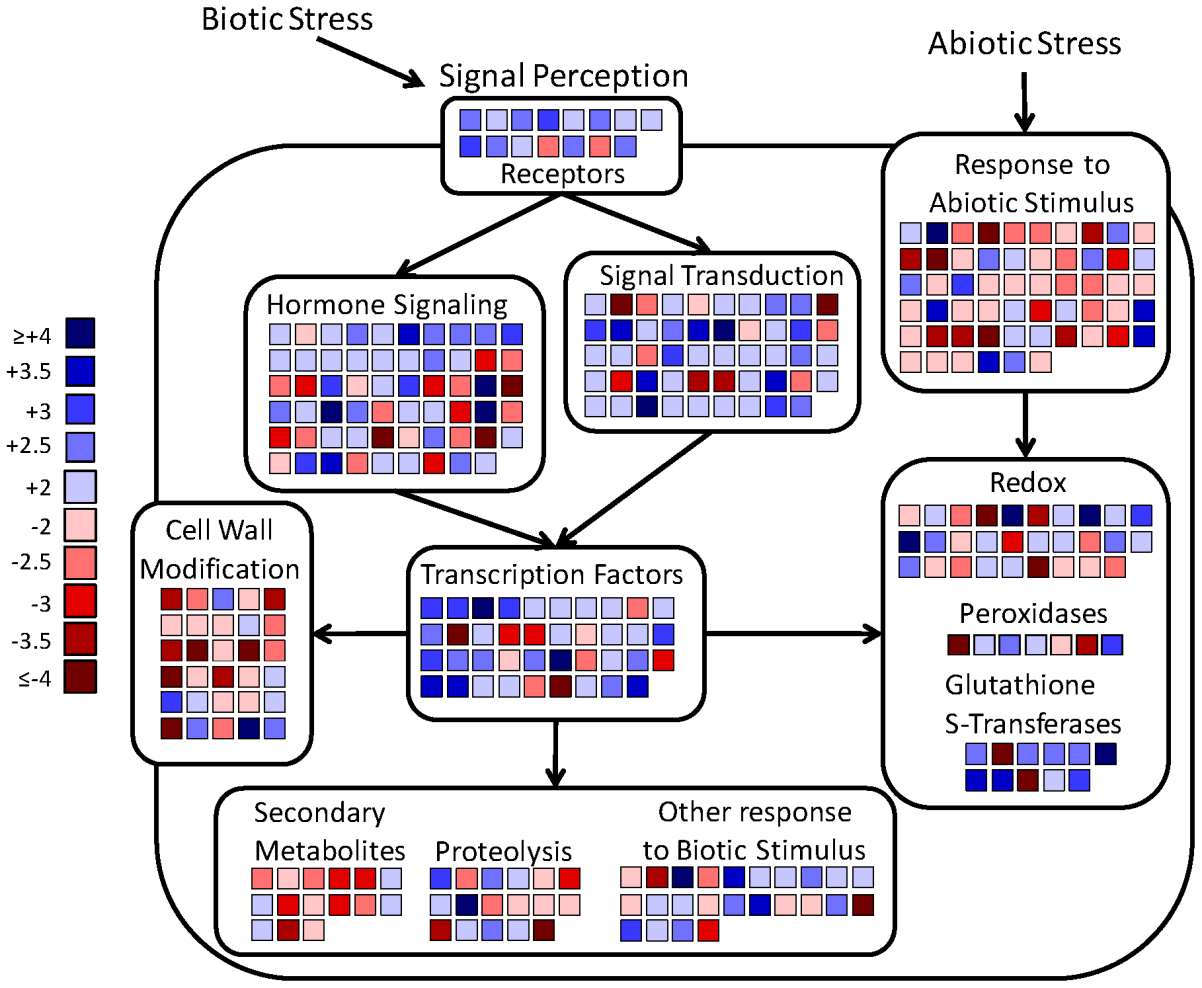
Schematic overview of genes exhibiting a change of 2-fold or more in response to melatonin associated with stress response and signaling. Fold change in transcript levels is represented by the color scale with darkest blue indicating genes with the greatest increase in transcript levels (4-fold or greater) and darkest red indicating genes with the greatest decrease in transcript levels (at least 4-fold). Each gene involved in stress responses is represented once.

Given that a large group of genes altered by melatonin are involved in plant stress tolerance, we further categorized these genes according to their specific roles in plant defense. A schematic of the plants response to biotic and abiotic stresses to include key events and players involved in perception, signaling, and counter-attack that were affected by melatonin is depicted in Figure 4. During biotic and abiotic stresses, stress perception involves transmembrane receptors which initiate a signal transduction cascade involving MAP Kinases mediated by transcription factors. A closer examination of stress-responsive genes indicates that melatonin altered the expression of stress-response genes involved in all the steps along the way: from receptors through transcription factors. In addition, hormonal signaling was also examined and included in the schematic (Figure 4) due to the structural similarity between melatonin and IAA and the cross-talk among hormonal pathways and their involvement in signaling during stress events.

### Melatonin-altered receptors, kinases, and calcium signaling genes pertaining to stress defense

When facing environmental stresses, plants first perceive stress signal and initiate a signal transduction cascade to defend against the pronounced stresses [75]–[77]. In order to understand the role of melatonin in plant stress defense, we identified all possible genes encoding receptors and kinases, and genes involved in calcium signaling with at least 2 fold changes in RNA-seq data. As shown in Table 5, all but 6 stress receptors identified exhibiting a change in transcript levels of at least 2 fold in response to melatonin were up-regulated. The plant immune response continued to trend towards up-regulation downstream of the receptors. The majority of kinases identified, including two MAPK and MAPKKK, were also up-regulated.

**Table 5:** Receptors, kinases, and genes involved in calcium signaling whose expression levels were affected by 1 mM melatonin by at least 2 fold.

Accession	Gene	Description	Fold Change
**Receptors**			
AT4G01870	AT4G01870	TolB related protein	3.18457
AT1G66090	AT1G66090	Disease resistance protein (TIR-NBS class)	3.16883
AT1G57630	AT1G57630	Toll-Interleukin-Resistance domain family protein	2.6298
AT4G14370	AT4G14370	TIR-NBS-LRR class disease resistance protein	2.42877
AT2G32660	RLP22	Receptor like protein 22	2.23431
AT2G31880	SOBIR1	Leucine rich repeat transmembrane protein	2.00079
**Kinases**			
AT1G66830	AT1G66830	Leucine-rich repeat receptor-like protein kinase	3.02283
AT1G51830	AT1G51830	Leucine-rich repeat protein kinase	2.84031
AT5G67080	MAPKKK19	Mitogen-activated protein kinase kinase kinase 19	2.67063
AT5G25930	AT5G25930	Leucine-rich repeat receptor-like protein kinase	2.53548
AT4G11890	ARCK1	Osmotic-stress-inducible receptor-like cytosolic kinase 1	2.50129
AT2G05940	RIPK	RPM1-induced protein kinase	2.40356
AT1G01560	MPK11	MAP Kinase 11	2.30442
AT4G04490	CRK36	Cysteine-rich receptor-like protein kinase	2.1158
AT1G21250	WAK1	Cell wall-associated kinase 1	2.05986
AT3G09010	AT3G09010	Protein kinase superfamily protein	2.02544
AT2G41820	AT2G41820	Leucine-rich repeat protein kinase family protein	−2.06899
AT5G04190	PKS4	Phytochrome kinase substrate 4	−2.76656
AT4G10390	AT4G10390	Receptor-like protein kinase	−3.10278
AT5G58300	MCK7	Leucine-rich repeat protein kinase family protein	Suppressed
**Genes involved in calcium signaling**			
AT2G25090	CIPK16	CBL-INTERACTING PROTEIN KINASE 16	4.56701
AT1G21550	AT1G21550	Calcium-binding EF-hand family protein	3.97178
AT1G74010	AT1G74010	Calcium-dependent phosphotriesterase superfamily	3.74794
AT3G22910	AT3G22910	calcium-transporting ATPase 13	3.34891
AT1G66400	CML23	Calmodulin-like protein 23	3.34393
AT5G26920	CBP60G	Calmodulin binding protein 60-like G	3.26761
AT1G76650	CML38	Calmodulin-like 38	3.18083
AT3G50360	CEN2	Centrin2; Involved in calcium ion binding	2.56703
AT3G47480	AT3G47480	Calcium-binding EF-hand family protein	2.29581
AT2G33380	RD20	Encodes a calcium binding protein	2.28141
AT5G63970	AT5G63970	Calcium-dependent phospholipid-binding protein	2.25577
AT3G22930	CML11	CALMODULIN-LIKE 11	2.08638
AT5G45820	CIPK20	CBL-interacting protein kinase 20	−3.86907
AT1G02900	RALF1	Similar to tobacco Rapid Alkalinization Factor (RALF)	−2.7376

Calcium signals are also a core regulator for plant cellular responses to environmental stimuli [78]–[80]. Interestingly, all but two of the 14 genes identified involving calcium-dependent signaling in our RNA-seq data were up-regulated in response to melatonin. These genes encode calcium binding protein, calmodulin like protein, CBL-interacting protein kinases and calcium dependent phosphotriesterase. The involvement of calcium signaling in melatonin-mediated pathways is extensively documented in mammalian system [81]–[83]. The discovery of similar components of this pathway in plants indicates that melatonin may use similar signaling to implement its critical role(s) in both plants and mammals.

### Transcription factors affected by melatonin

A total of 53 transcription factors were identified with at least 2 fold changes by melatonin treatment (Table 6). Interestingly, all but one of the 29 transcription factors identified as up-regulated genes are stress related transcription factors. These genes include 8 WRKY transcription factors, 5 NAC domain containing proteins and 5 zinc finger related transcription factors. The ERF transcription factor, CEJ1, is also up-regulated. CEJ1 mediates immune responses in Arabidopsis by repressing DREB [84]. In the melatonin down-regulated transcription factor group, 16 of the 24 genes are related to stress responses including 5 ERF (ethylene response factor) transcription factors. Two bZIP transcription factors are also partially suppressed by melatonin.

**Table 6:** Transcription factors with changes in gene expression levels of at least 2 fold following 1 mM Melatonin treatment.

Accession #	Gene	Description	Fold change	stress
AT4G22070	WRKY31	WRKY DNA-binding protein 31	4.04382	**+**
AT1G02450	NIMIN1	NIM1-Interacting 1	4.01997	**+**
AT5G64810	WRKY51	WRKY DNA-binding protein 51	3.89444	**+**
AT5G26170	WRKY50	WRKY DNA-binding protein 50	3.60762	**+**
AT5G26920	CBP60G	Calmodulin binding protein 60-like.g	3.51898	**+**
AT3G46080	AT3G46080	C2H2-type zinc finger family protein	3.443	**+**
AT3G46090	ZAT7	Zinc finger protein	3.36216	**+**
AT1G02580	MEA	Maternal embryogenesis control protein	3.23147	
AT1G01720	NAC002	NAC domain containing protein 2	3.07132	**+**
AT1G01010	NAC001	NAC domain containing protein 1	3.03835	**+**
AT3G56400	WRKY70	WRKY DNA-binding protein 70	2.74608	**+**
AT4G01250	WRKY22	WRKY DNA-binding protein 22	2.73313	**+**
AT2G38250	AT2G38250	Homeodomain-like superfamily protein	2.6849	**+**
AT5G63790	NAC102	NAC domain containing protein 102	2.60489	**+**
AT3G50260	CEJ1	Cooperatively regulated by ethylene and jasmonate 1	2.57229	**+**
AT5G13080	WRKY75	WRKY DNA-binding protein 75	2.53783	**+**
AT1G73805	SARD1	SAR Deficient 1	2.41244	**+**
AT5G59820	RHL41	Zinc finger protein	2.39584	**+**
AT1G62300	WRKY6	WRKY DNA-binding protein 6	2.37767	**+**
AT5G01380	AT5G01380	Homeodomain-like superfamily protein	2.28926	**+**
AT2G16720	MYB7	MYB domain protein 7	2.26732	**+**
AT3G19580	AZF2	Zinc finger protein 2	2.20418	**+**
AT5G49450	bZIP1	Basic leucine zipper 1	2.1947	**+**
AT1G52890	NAC019	NAC domain-containing protein 19	2.19063	**+**
AT5G62020	HSFB2A	Heat shock transcription factor B2A	2.10743	**+**
AT3G04070	NAC047	NAC domain containing protein 47	2.06633	**+**
AT1G27730	STZ	Salt tolerance zinc finger	2.05565	**+**
AT2G40750	WRKY54	WRKY DNA-binding protein 54	2.05047	**+**
AT3G24500	MBF1C	Multiprotein bridging factor 1C	2.04727	**+**
AT5G20240	PI	Floral homeotic protein PISTILLATA	−2.00396	
AT3G02380	COL2	Zinc finger protein CONSTANS-LIKE 2	−2.16211	**+**
AT3G58120	BZIP61	Basic-leucine zipper transcription factor family protein	−2.18023	**+**
AT1G76880	AT1G76880	Duplicated homeodomain-like superfamily protein	−2.24420	**+**
AT1G35560	TCP23	TCP domain protein 23	−2.26984	**+**
AT3G10040	AT3G10040	Sequence-specific DNA binding transcription factor	−2.41108	**+**
AT1G75240	HB33	Homeobox protein 33	−2.51608	**+**
AT5G05790	AT5G05790	Homeodomain-like superfamily protein	−2.57254	
AT1G14600	AT1G14600	Homeodomain-like superfamily protein	−2.60754	
AT1G09530	PAP3	Phytochrome-associated protein 3	−2.67198	**+**
AT4G32800	AT4G32800	Ethylene-responsive transcription factor ERF043	−2.85108	**+**
AT5G60890	MYB34	Myb-like transcription factor	−2.9674	**+**
AT2G44940	AT2G44940	Ethylene-responsive transcription factor ERF034	−3.01312	**+**
AT4G17810	AT4G17810	C2H2 and C2HC zinc finger-containing protein	−3.05949	**+**
AT2G42380	BZIP34	BZIP family of transcription factors	−3.10503	**+**
AT1G19510	ATRL5	RAD-like 5	−3.32387	
AT1G04250	AXR3	Auxin resistant 3	−3.49394	**+**
AT5G25190	ESE3	Ethylene-responsive transcription factor ERF003	−3.49854	**+**
AT5G56840	AT5G56840	myb-like transcription factor family protein	−3.52364	
AT1G04240	SHY2	Short hypocotyl 2	−3.70592	
AT2G39250	SNZ	AP2-like ethylene-responsive transcription factor	−4.03198	**+**
AT5G61890	AT5G61890	Ethylene-responsive transcription factor ERF114	−4.23551	**+**
AT1G32360	AT1G32360	CCCH-type Zinc finger family protein	−4.39267	
AT2G18328	RL4	RAD-like 4	−4.82888	

### Melatonin altered gene expression along stress-induced hormone signaling pathways

Plant hormones and their cross-talk play important roles in both biotic and abiotic stress defense. Of these, abscisic acid (ABA) and ethylene (ET) are known for their abiotic stress defense functions [85]–[87], while salicylic acid (SA) and jasmonic acid (JA) are critical for plant biotic stress defense [88]–[90]. In addition, indole acetic acid (IAA) or auxin, also functions in plant stress defense systems [91]–[92]. In light of these stress-induced hormone signals, we identified 183 genes involved in hormone signaling exhibiting at least a 2-fold change in transcript levels in response to melatonin (Table S5). Of these, 52 genes pertaining to auxin responses and signaling were altered by melatonin with 29 down-regulated and 23 up-regulated. Most of the auxin responsive genes that were down-regulated in response to melatonin are involved in Auxin transport and homeostasis. Furthermore, one of the up-regulated genes encodes a GH3 protein, an IAA-amino synthase which conjugates amino acids to IAA, thus inactivating it [93]. These results may suggest that the Arabidopsis seedlings were responding to high levels of melatonin as they would respond to excess auxin.

Two ACC synthases were also up-regulated in response to melatonin, suggesting that melatonin may induce ethylene biosynthesis. One of the ACC synthases identified is ACS8, an auxin inducible ACC synthase. Indeed, this response is similar to what was induced by 2,4-D [94] and consistent with defense responses in plants. However, none of the known genes on auxin biosynthesis pathways [95] were significantly altered in their expression in response to melatonin (Table S3). This result is in agreement with findings that auxin inducible DR5:GUS reporter gene cannot be induced by melatonin and the function of melatonin is independent of auxin [60].

While the majority of auxin-responsive genes were down-regulated in response to 1 mM melatonin, most genes on ABA, SA, JA and ET pathways were up-regulated (Table S5). On the ABA pathway, 36 out of 50 genes were up-regulated, 70 out of 92 were up-regulated on the SA pathway, 53 out of 67 were up-regulated on the JA pathway, and 32 out of 42 were up-regulated on the ET pathway. As expected, some of these genes are induced by multi-hormone signals confirming important roles of crosstalk among hormones in plant defense systems. Many of the SA, JA, ABA and ET responsive genes induced by melatonin are also induced in response to biotic and abiotic stresses. Consistent with the changes in upstream gene expression levels, many downstream stress-associated genes were also affected, as shown in Table S6. These results further confirm the critical roles of melatonin in defense against both biotic and abiotic stresses in plants.

### Most cell wall associated genes were down-regulated by melatonin

Genes with functions involving the cell wall, including modification and growth, were largely affected by melatonin. Of the 60 genes associated with the cell wall, 45 were down-regulated and 14 were up-regulated at least 2-fold, and one gene was completely suppressed (Table 7). The down-regulated genes include 8 expansions and 4 pectin lyases or pectin methyltransferases. Two xyloglucan endotransglucosylases (XTH) were identified among up-regulated genes.

**Table 7:** Cell wall related genes with at least a 2 fold change in expression levels in response to 1 mM Melatonin.

Accession #	Gene	Description	Fold Change
AT4G25810	XTR6	Xyloglucan endotransglycosylase 6	4.46599
AT5G57550	XTH25	Xyloglucan endotransglucosylase/hydrolase 25	4.05392
AT3G60140	SRG2	Beta-glucosidase 30	3.32985
AT2G45220	AT2G45220	Plant invertase/pectin methylesterase inhibitor superfamily	2.85982
AT1G76470	AT1G76470	NAD(P)-binding Rossmann-fold superfamily protein	2.75965
AT5G57560	TCH4	Xyloglucan endotransglucosylase/hydrolase 22	2.67655
AT4G30280	XTH18	Xyloglucan endotransglucosylase/hydrolase 18	2.57069
AT1G21670	AT1G21670	uncharacterized protein	2.46195
AT4G01430	UMAMIT29	Nodulin MtN21-like transporter family protein	2.42787
AT1G78770	APC6	Anaphase promoting complex 6	2.35509
AT3G45970	ATEXPL1	Expansin-like A1	2.12646
AT4G26470	CML21	Calmodulin-like protein 21	2.10667
AT1G05560	UGT75B1	UDP-glucosyltransferase 75B1	2.08708
AT2G43570	CHI	Putative chitinase	2.04539
AT2G37040	PAL1	Phenylalanine ammonia-lyase 1	−2.04568
AT4G08685	SAH7	Allergen-like protein	−2.07476
AT2G39700	EXPA4	Alpha-Expansin 4	−2.09564
AT5G14610	AT5G14610	DEAD box RNA helicase family protein	−2.10274
AT3G19450	ATCAD4	Cinnamyl alcohol dehydrogenase 4	−2.11144
AT1G70370	PG2	Polygalacturonase 2	−2.11182
AT4G33220	PME44	Pectin methylesterase 44	−2.13826
AT2G33330	PDLP3	Plasmodesmata-located protein 3	−2.15409
AT2G36870	XTH32	Xyloglucan endotransglucosylase/hydrolase 32	−2.22347
AT3G26520	SITIP	Gamma-tonoplast intrinsic protein 2	−2.22602
AT1G03630	PORC	Protochlorophyllide oxidoreductase C	−2.23808
AT2G45470	FLA8	Fasciclin-like arabinogalactan protein 8	−2.26278
AT4G36360	BGAL3	Beta-galactosidase 3	−2.27535
AT1G24100	UGT74B1	UDP-glucosyl transferase 74B1	−2.28945
AT1G69530	EXPA1	Alpha-Expansin 11	−2.31131
AT5G20540	BRXL4	Brevis radix-like 4	−2.32922
AT5G47500	PME5	Pectin methylesterase 5	−2.38968
AT4G12880	ENODL19	Early nodulin-like protein 19	−2.40384
AT5G04360	PU1	Pullulanase 1	−2.41719
AT1G05850	ELP	Endo chitinase-like protein	−2.43396
AT3G44990	XTH31	Xyloglucan endo-transglycosylase	−2.44701
AT5G53090	AT5G53090	NAD(P)-binding Rossmann-fold superfamily protein	−2.68485
AT1G04680	AT1G04680	Pectin lyase-like superfamily protein	−2.68802
AT5G48900	AT5G48900	Pectin lyase-like superfamily protein	−2.69330
AT3G05900	AT3G05900	Neurofilament protein-related	−2.78189
AT2G28950	EXPA6	Alpha-Expansin 6	−2.78667
AT3G16370	AT3G16370	GDSL-like Lipase/Acylhydrolase superfamily protein	−2.83692
AT5G03760	ATCSLA09	Cellulose synthase like A9	−2.88537
AT4G39330	CAD9	Cinnamyl alcohol dehydrogenase 9	−2.991
AT2G19590	ACO1	1-aminocyclopropane-1-carboxylate oxidase 1	−3.13848
AT2G34410	RWA3	Reduced wall acetylation 3	−3.20576
AT3G29030	EXPA5	Alpha-Expansin 5	−3.25293
AT1G04040	AT1G04040	HAD superfamily, subfamily IIIB acid phosphatase	−3.32924
AT2G37640	EXP3	Alpha-Expansin 3	−3.40584
AT1G20190	EXPA11	Alpha-Expansin 11	−3.47301
AT5G19730	AT5G19730	Pectin lyase-like superfamily protein	−3.62317
AT1G43800	FTM1	Floral transition at the meristem 1	−3.98758
AT4G22610	AT4G22610	Protease inhibitor/seed storage/lipid transfer family protein	−4.01733
AT4G28250	EXPB3	Beta-expansin/allergen protein.	−4.03468
AT3G10340	PAL4	Phenylalanine ammonia-lyase	−4.14771
AT2G38120	AUX1	Auxin influx transporter	−4.28187
AT3G23090	WDL3	Targeting protein for Xklp2 protein family	−4.66044
AT2G40610	EXPA8	Alpha-Expansin Gene Family	−4.89447
AT4G02290	GH9B13	Glycosyl hydrolase 9B13	−5.14512
AT1G26250	AT1G26250	Proline-rich extensin-like family protein	−7.28679
AT4G31850	PGR3	Pentatricopeptide repeat-containing protein	suppressed

The results in the current study are significantly different from our previous study in cucumber roots showing that many of cell wall related genes, particularly pectinesterase genes, were up-regulated by melatonin [66]. These discrepancies could be explained by different species and tissues being used or the dissimilar methods of melatonin treatment between the two studies. Therefore, it is necessary to further examine the role(s) of these cell wall related genes in melatonin-mediated signaling pathway. In general, cell wall modification and strengthening is the first line of defense plants have against pathogens [96]–[97]. Plant growth involving cell wall expansion would make cells more vulnerable to pathogen attack. Furthermore, XTHs are involved in strengthening the cell wall [98]. Thus, in the event of biotic stress, it is beneficial for plants to suppress cell wall expansion while strengthening the cell wall via XTHs. As a result, the cell wall will be more difficult for pathogens to breach. These discoveries are consistent with the studies reporting the role of melatonin in plant disease resistance [52].

### Melatonin alters expression of redox associated genes

Genes and their protein products involved in various redox pathways are also used to protect cells from oxidative damage [99]–[100]. Many genes related to redox homeostasis were identified among the transcripts that were significantly differentially expressed when treated with melatonin (Table 8). In total, we identified 59 genes involved in redox homeostasis with significant changes in expression. Of these, 36 were down-regulated, and 23 were up-regulated by melatonin treatment. Most genes (53 of 59) identified were related to biotic and/or abiotic stress responses. Several members of the same families were differently affected by melatonin. For example, 7 of the 10 cytochromes, 6 of the 9 reductases, 4 of the 5 oxygenases, and 3 of the 5 oxidases were down-regulated by melatonin.

**Table 8:** Redox-related genes and peroxidases affected by 1 mM melatonin.

Accession #	Gene	Description	Fold change	Stress response
**Redox-Related Genes**				
AT2G26400	ATARD3	Acireductone dioxygenase 3	4.84062	**+**
AT5G56970	CKX3	Cytokinin oxidase 3	4.47185	**+**
AT3G04000	AT3G04000	Aldehyde reductase	4.39375	**+**
AT2G37770	ChlAKR	Chloroplastic aldo-keto reductase	4.27787	**+**
AT5G48450	SKS2	SKU5 Similar	4.01997	
AT3G21460	AT3G21460	Glutaredoxin family protein	3.82732	
AT1G04580	AAO4	Aldehyde oxidase 4	3.27115	**+**
AT2G34500	CYP710A1	Cytochrome P450 710A1	3.09868	**+**
AT5G07440	GDH2	Glutamate dehydrogenase 2	2.99839	**+**
AT1G76470	AT1G76470	NAD(P)-binding Rossmann-fold superfamily protein	2.75965	**+**
AT1G30700	AT1G30700	FAD-binding Berberine family protein	2.73673	**+**
AT2G47130	SDR3	short-chain dehydrogenase/reductase 2	2.6147	**+**
AT4G34120	CDCP1	Cystathione [Beta]-synthase domain-containing protein 1	2.52354	**+**
AT1G54100	ALDH7B4	aldehyde dehydrogenase 7B4	2.42985	**+**
AT4G20830	AT4G20830	FAD-binding Berberine family protein	2.42483	**+**
AT1G26390	AT1G26390	FAD-binding Berberine family protein	2.40531	**+**
AT4G20860	AT4G20860	FAD-binding Berberine family protein	2.31804	**+**
AT3G14620	CYP72A8	Cytochrome P450 72A8	2.24781	**+**
AT4G37990	ATCAD8	Arabidopsis thaliana cinnamyl-alcohol dehydrogenase 8	2.23169	**+**
AT1G09500	AT1G09500	Alcohol dehydrogenase-like protein	2.1254	**+**
AT1G30730	AT1G30730	FAD-binding Berberine family protein	2.0479	**+**
AT5G24530	DMR6	Downy mildew resistant 6	2.01769	**+**
AT3G26210	CYP71B23	Cytochrome P45071B23	2.01045	**+**
AT2G32720	CB5-B	Cytochromes B5	−2.11096	**+**
AT3G19450	ATCAD4	Cinnamyl alcohol dehydrogenase 4	−2.11144	**+**
AT4G00360	CYP86A2	Cytochrome P450 86A2	−2.16096	**+**
AT1G14345	AT1G14345	NAD(P)-linked oxidoreductase superfamily protein	−2.16512	**+**
AT2G46750	GulLO2	L -gulono-1,4-lactone oxidase 2	−2.16775	**+**
AT1G03630	PORC	Protochlorophyllide oxidoreductase C	−2.23808	**+**
AT5G43750	NDH18	NAD(P)H dehydrogenase 18	−2.26847	**+**
AT5G49730	ATFRO6	Ferric reduction oxidase 6	−2.35762	**+**
AT5G07460	ATMSRA2	Methionine sulfoxide reductase 2	−2.38061	**+**
AT1G14150	PnsL2	Photosynthetic NDH subcomplex L 2	−2.39048	**+**
AT1G17650	GLYR2	Glyoxylate reductase 2	−2.46655	**+**
AT1G07440	AT1G07440	NAD(P)-binding Rossmann-fold superfamily protein	−2.56359	**+**
AT5G14200	ATIMD1	Arabidopsis isopropylmalate dehydrogenase 1	−2.62532	**+**
AT1G62540	FMO GS-OX2	Flavin-monooxygenase glucosinolate S-oxygenase 2	−2.65092	**+**
AT2G39470	PPL2	Photosynthetic NDH subcomplex L 1	−2.6738	**+**
AT5G53090	AT5G53090	NAD(P)-binding Rossmann-fold superfamily protein	−2.68485	
AT5G44410	AT5G44410	FAD-binding Berberine family protein	−2.79494	**+**
AT4G25100	FSD1	FE-superoxide dismutase 1	−2.79727	**+**
AT4G39330	CAD9	Cinnamyl alcohol dehydrogenase 9	−2.991	**+**
AT5G58260	NDHN	NADH Dehydrogenase-like complex N	−2.99281	**+**
AT4G25600	AT4G25600	Oxoglutarate/iron-dependent oxygenase	−3.07505	
AT2G19590	ACO1	1-aminocyclopropane-1-carboxylate oxidase 1	−3.13848	**+**
AT4G39510	CYP96A12	Cytochrome P450 96A12	−3.2424	**+**
AT1G65860	FMO GS-OX1	Flavin-monooxygenase glucosinolate S-oxygenase 1	−3.3226	**+**
AT1G06350	ADS4	Fatty acid desaturase family protein	−3.33981	**+**
AT3G01440	PnsL3	Photosynthetic NDH subcomplex L 3	−3.34393	**+**
AT4G13770	CYP83A1	Cytochrome P450 83A1	−3.3894	**+**
AT4G25310	AT4G25310	2-oxoglutarate and Fe(II)-dependent oxygenase	−3.84556	**+**
AT4G19380	AT4G19380	Long-chain fatty alcohol dehydrogenase family protein	−3.84622	
AT1G43800	FTM1	Stearoyl-acyl-carrier-protein desaturase family protein	−3.98758	**+**
AT1G16400	CYP79F2	Cytochrome P450 79F2	−4.03821	**+**
AT5G42800	DFR	Dihydroflavonol 4-reductase	−4.19704	**+**
AT4G12320	CYP706A6	Cytochrome P450 706A6	−4.31681	**+**
AT1G16410	CYP79F1	Cytochrome P450 79F1	−4.38148	**+**
AT1G30760	AT1G30760	FAD-binding Berberine family protein	−4.5319	**+**
AT2G18030	MSRA5	Methionine sulfoxide reductase A5	−5.04612	
**Peroxidase Genes**				
AT5G39580	AT5G39580	Peroxidase superfamily protein	3.33272	**+**
AT1G14550	AT1G14550	Peroxidase superfamily protein	2.66383	**+**
AT1G14540	PER4	Peroxidase 4	2.46391	**+**
AT3G49120	PRXCB	Peroxidase CB	2.45042	**+**
AT4G33420	AT4G33420	Peroxidase superfamily protein	2.2565	**+**
AT3G21770	AT3G21770	Peroxidase superfamily protein	−2.21265	**+**
AT4G08770	AT4G08770	Encodes a putative apoplastic peroxidase Prx37	−2.21812	**+**
AT3G26060	PRXQ	Peroxiredoxin Q	−2.31392	**+**
AT4G08780	AT4G08780	Peroxidase superfamily protein	−2.99013	**+**
AT1G49570	AT1G49570	Peroxidase superfamily protein	−3.03705	**+**
AT4G30170	AT4G30170	Peroxidase family protein	−3.79494	**+**
AT1G05260	RCI3	Cold-inducible cationic peroxidase	−4.07505	**+**
AT4G11290	AT4G11290	Peroxidase superfamily protein	−4.97627	**+**

Peroxidases comprise another group of genes related to oxidative stress and are used as biochemical markers for plant resistance to bacterial and fungal diseases [101]–[103]. Thirteen genes encoding peroxidases were identified with significantly different expression levels following melatonin treatment (Table 8). Eight of these genes were down-regulated by melatonin and five were up-regulated. All identified peroxidase genes were reported to be associated with plant stress defense. Of these, 8 (At1g05260, At1g14540, At3g21770, At3g49120, At4g08770, At4g08780, At4g12290, and At5g39580) were linked with plant biotic stress defense [104]–[106] and 5 (At1g05260, At1g14550, At1g49570, At4g30170, and At4g33420) were involved in plant abiotic stress defense [107]–[108]. The trend towards down-regulation of peroxidase genes in the current study is also contradictory to our previous study using cucumber roots [66] where most peroxidase genes were up-regulated by melatonin. Comparative analysis between these two studies indicates that most peroxidase genes identified in these studies belong to different members of the same gene family, with only two genes [At1G49570 (PER10), and At4G11290 (PER39)] mutual to both studies. Peroxidases belong to a large gene family with more than 70 members in plant species [109]. Their expression levels and patterns in different tissues vary among tissues [109] indicating they play important roles in a diversity of developmental processes [110]–[111].

### Melatonin alleviates paraquat-induced photobleaching

**Figure 5:**
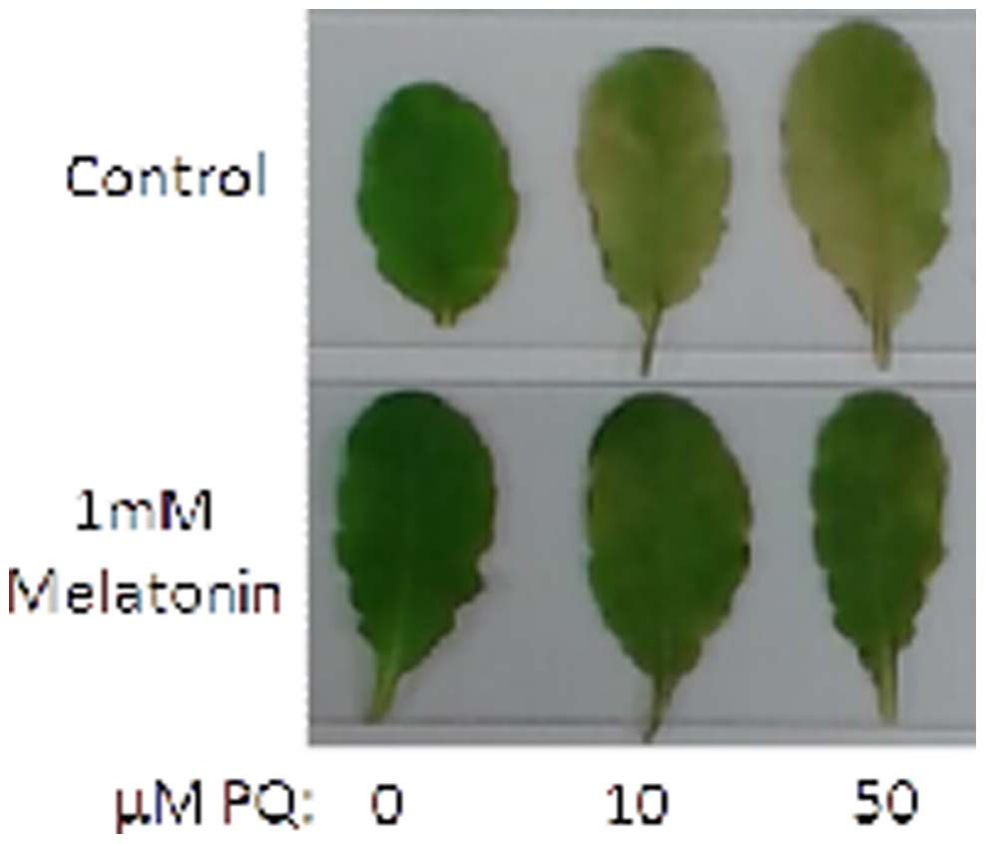
Effect of melatonin on paraquat-induced oxidative stress. Arabidopsis leaves were detached and floated in solution containing 0, 10(top row) or presence (bottom row) of 1 mM melatonin. After 48 hours, leaves exposed to paraquat in the absence of melatonin were photobleached while leaves incubated with melatonin during exposure to paraquat remained green.

Since the majority of genes with altered expression levels are involved in plant stress defense and melatonin is a potent antioxidant, we further examined the potential of melatonin to alleviate paraquat induced oxidative stress. Four-week old detached Arabidopsis leaves were incubated in 0 mM, 10 mM or 50 mM paraquat in the presence or absence of 1 mM melatonin. Leaves treated with paraquat in the absence of melatonin were completely photobleached after 48 hours under 16/8 hour light/dark photoperiod (Figure 5). Leaves treated with 1 mM melatonin remained green, similar to leaves in the absence of paraquat. This result clearly demonstrated that melatonin can play critical roles in attenuating oxidative stress in plants. Since melatonin is able to alleviate paraquat-induced disease in mammals [28]–[30], a similar role(s) of melatonin in defense against oxidative stresses may exist in both animals and plants. We can further utilize Arabidopsis as a model to elucidate the mode of action melatonin employs to protect against oxidative stress-induced diseases in humans and to further our understanding of its unique role(s) in plant species.

## Conclusion

In conclusion, we report here the first comprehensive analysis of the effect of melatonin on genome-wide gene expression in Arabidopsis seedlings using RNA-seq technology. Given that Arabidopsis is an established plant model species and forward and reverse genetics methodologies are readily available, these datasets will provide fundamental information and serve as new tools to genetically dissect melatonin-mediated pathway(s) either common to both plants and animals, or unique to plants. Our transcriptome analysis reveals broader roles of melatonin in regulating plant growth and development. However, more importantly, melatonin may play critical role(s) in plant defense systems. Out of nearly 900 genes that were significantly up- or down- regulated by melatonin with at least 2 fold changes, almost 40% of the genes were related to plant stress defense, including many stress receptors, kinases, and transcription factors, as well as downstream genes encoding end products that were directly used for stress defense. Furthermore, the expression of many genes involved in different hormone signaling pathways such as auxin, ABA, SA, ET and JA, and linked to plant stress defense, was also altered in response to melatonin treatment. Concurrently, expression of many cell wall associated genes, and genes involved in redox pathways, particularly peroxidases were significantly changed by melatonin treatments. Taken together, our results suggest that melatonin plays a critical role in plant defense against environmental stresses, including both biotic and abiotic stresses. Further dissection of the melatonin mediated pathway may lead to the development of novel strategies for crop improvement in the face of ubiquitous environmental stresses.

## Supporting Information

Figure S1 Scatter plots between treatments.
(TIF)

Figure S2 Phylogenetic analysis of all clean RNA-seq data from six constructed cDNA libraries.
(TIF)

Table S1 List of genes and their primer pairs used for qRT-PCR validation.
(DOCX)

Table S2 List of genes that are significantly affected by 100 pM melatonin.
(DOCX)

Table S3 List of genes that are significantly affected by 1 mM melatonin.
(DOCX)

Table S4 qRT-PCR validation of RNA-seq data.
(DOCX)

Table S5 Genes with changes in expression levels of at least 2 fold in response to 1 mM Melatonin involved in one or more hormone signaling pathways.
(DOCX)

Table S6 Downstream genes in plant stress defense that are affected by melatonin and their fold changes.
(DOCX)
