# Supplementary figures and images for: Arabidopsis Transcriptome Analysis Reveals Key Roles of Melatonin in Plant Defense Systems
Source: PLoS One. 2014 Mar 28;9(3):e93462. doi: 10.1371/journal.pone.0093462 (PMC3969325; doi:10.1371/journal.pone.0093462)

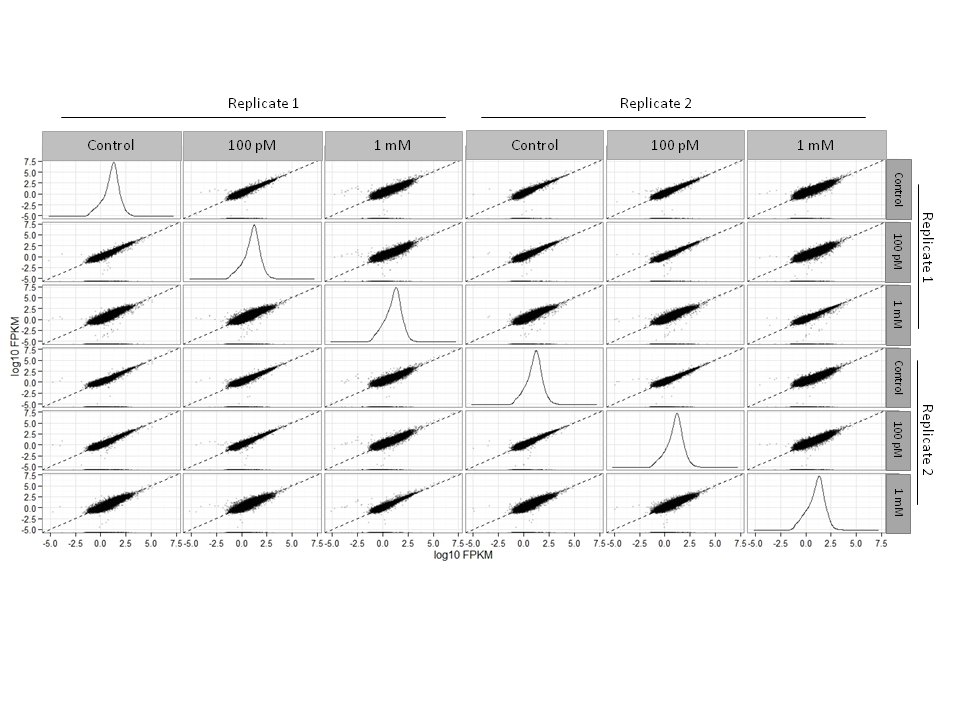

Supplement: Figure S1 — Scatter plots between treatments. (TIF) [file pone.0093462.s001.tif]

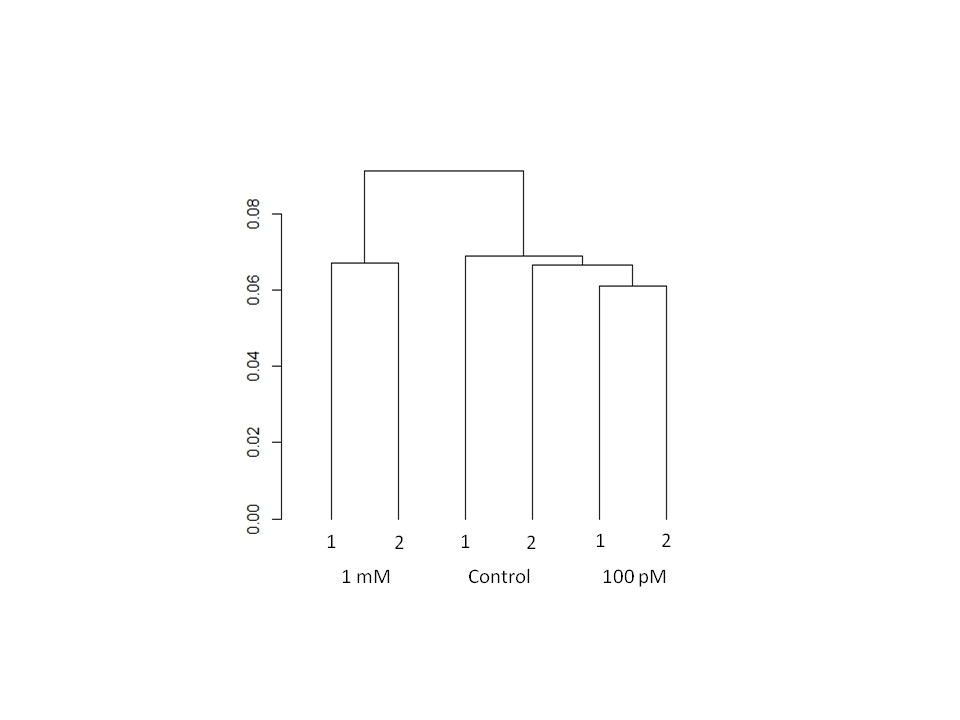

Supplement: Figure S2 — Phylogenetic analysis of all clean RNA-seq data from six constructed cDNA libraries. (TIF) [file pone.0093462.s002.tif]
