# Supplementary material for: Arabidopsis Transcriptome Analysis Reveals Key Roles of Melatonin in Plant Defense Systems
Source: PLoS One. 2014 Mar 28;9(3):e93462. doi: 10.1371/journal.pone.0093462 (PMC3969325; doi:10.1371/journal.pone.0093462)
Supplement: Table S1 — List of genes and their primer pairs used for qRT-PCR validation. (DOCX) [file pone.0093462.s003.docx]

**Table S1:** List of genes and their primer pairs used for qRT-PCR validation.

| Accession # | Gene | Forward Primer | Reverse Primer |
| --- | --- | --- | --- |
| AT1G01310 | CAP | GACAGGCGTAGCAGCAGTTG | GCACATCCCAACCTCTCCG |
| AT1G02450 | NIMIN1 | CAAGAAGCACGGAAACGTAGAC | GTACGACACTGAGAGAAATCCTC |
| AT1G04250 | AXR3 | GGCAAACATGGAGGAGAAGAAG | CGACGAGCATCCAATCACCG |
| AT1G05560 | UGT75B1 | CAAGGATGGTTTGGTGGAGAG | CATTTCTTTGCGTTTTCCCTCAAC |
| AT1G19670 | ATCLH1 | CGGTTGTATGTGTAAGAATGGG | CCACAAACTATACTTGAGAAACG |
| AT1G28290 | AGP31 | CCTAAGACGGTGACGAACTTC | CGACATCTCCACCGAAGAGC |
| AT1G54040 | ESP | GCTGTCCAATGAGGGATATGC | CTCGTGGTATGGCTGGTGC |
| AT1G54100 | ALDH7B4 | CACTCGGAACCCTGAAAACATC | CAGCTCCATTCGTCGGTATG |
| AT1G63840 | AT1G63840 | CGAGAACGACGACGAGATCC | GTGTACGACAAAGTGGACACG |
| AT1G73805 | SARD1 | GAAGTGGGCGAACGAACAGC | GTAGTGGCTCGCAGCATATTG |
| AT1G74020 | SS2 | CAAAGCTGGTTCTTCTGAAGAC | GTACGATGATCTTGTTCACCAC |
| AT1G74670 | GASA6 | GTGGAGGACAATGCACAAGG | GCCTGGAGGGACACAAAGG |
| AT1G76650 | CML38 | GGAGCGTTTAGACTGTATATTGC | AGTGGTTCTTGATTCTCCTAGC |
| AT2G14610 | PR1 | GAGAAGGCTAACTACAACTACG | CACCTCACTTTGGCACATCC |
| AT2G23170 | GH3.3 | GCAGAGACGAAGACTATACCTG | GTTCAACGACTCCTCCATTTCC |
| AT2G25090 | CIPK16 | GTTGGAGTATTACCTCTTCTGTG | CATCACCTTGCCATGACCATAC |
| AT2G26020 | PDF | CATCACCCTTATCTTCGCTGC | CATGTCCCACTTGGCTTCTC |
| AT2G27150 | AAO3 | GGAAGTGGACCTTGTGACAG | CCTTGAACAAATGCTCCTTCG |
| AT2G29420 | ATGSTU7 | CGTCGATGAACAGATTTATGTGAC | CTTTCCCAACAAGCTCTTTCTCC |
| AT2G29490 | ATGSTU1 | CTTCCTCAAGATCCTTACGAG | CAACCTCTCTTCCTTTCTCTG |
| AT2G32140 | AT2G32140 | CTACCGATATGTACGATACACC | CCG ACT CCG ACT CCA TTG TTG |
| AT2G33380 | RD20 | TGGAATGTAACCGAGGGAAATCG | GAAACCATCTTCGTCCTTAGCAAG |
| AT2G36800 | DOGT1 | GCTGGTCTACCGCTACTTAC | CCATTTCATAGGCTGTTCAACC |
| AT2G38210 | PDX1L4 | GGAGTTGTGGCGGTGTACG | CCACGAAGCATCTGAGCGAG |
| AT2G40000 | HSPRO2 | GGAGGACCAAGATTACAAGAGG | GCATCCCCTGAAGCAAATGAATC |
| AT2G41230 | ORS1 | GATTCTGCCACCGTTCCTGC | GAGGCTGGGCTCATTAGAAG |
| AT3G04290 | LTL1 | CAGCCAATGCCTATCAAATGAAC | GTACGGTCCTTGTCCACAAC |
| AT3G11480 | BSMT1 | CATGCCGTTTTATGATCCGAACG | CATTGCGTCCTGCTTCAAAGTC |
| AT3G12830 | AT3G12830 | CGAGATGGAGAGGTTCGTCG | CCATACTCTTGAGCAGATCGG |
| AT3G20470 | GRP5 | GGTTCAGGTGGAGGGTTTGG | CCGCTTCCGCCTCCTCC |
| AT3G22060 | AT3G22060 | CATTCAACTCGCAGACGAAAGC | CCGATATTTTTCTCTCCTGTCGC |
| AT3G22231 | PCC1 | TTGGTTATCCGACTAGAGATGC | GCAGAAGATACACTCCATACAAG |
| AT3G28210 | PMZ | GAGATTCTGACTTTTGCGAATAACC | GACATGATCTGTCGGAAACCG |
| AT3G28220 | AT3G28220 | GGCGAAGGACAGGGTAACTC | GTTTGCTTGATCCGACCAGC |
| AT3G49780 | ATPSK4 | CCAGAGAAAACTCCGTCAAGG | CATTCTTCTTCTCCAATTCCGTTG |
| AT3G52400 | SYP122 | GATATGGCTGTGTTGGTTGAG | CAAAATGGCAAAGCAAGTCCAC |
| AT3G55970 | JRG21 | GTATCTCTCCGCACTCTGATC | CGACTGTGATCCAAGCGTC |
| AT3G57260 | BGL2 | GAATGGATCACCGAGAAGGC | CGATCTGGATGAAACAGTCCC |
| AT4G08950 | EXO | GCAACGAACCCTTTTGGTAATGG | CCAGGACAAGCAGAAGCAGC |
| AT4G13770 | CYP83A1 | TCAAAGGCACGGACTACGAG | GAGGAGAAGGTTCGCATAAGG |
| AT4G15390 | AT4G15390 | GTTTGGGTTGTTGGGAATGTGTC | GGCAGTGTGACGAACGCTTC |
| AT4G16740 | ATTPS03 | GCTCAGTGCGTTTATCAGTAC | GTGGAAGAGGGTGGACGAG |
| AT4G23810 | WRKY53 | CAGAGTCAAACCAGCCATTAC | CGTATCAGGGAACGAGAAAAC |
| AT4G25100 | FSD1 | CCTTGTGCTCGGCTCTTTCC | GTAATCTGGTCTTCGGTTCTGG |
| AT4G26200 | ACS7 | GAACGCAGGGCTATTTTGTTGG | GATCACATCCCAAAGCTGGAG |
| AT4G34138 | UGT73B1 | CGTTATTAGAAGGGGTGGCAG | CACTCCCACACTCACTCCTG |
| AT4G39030 | EDS5 | GTTTACTCGGCTGCTGGTTC | GTATGCCTCCAGGCGAAAG |
| AT5G07010 | ST2A | GTTGGGATACTGGAGAGAGAG | CAAGCCTCTTCAAGTTGGTCTC |
| AT5G13190 | GILP | CTCAGATCGAAACCTGGTGTAG | CAATGGTGCTGTTTGTTCCAGAG |
| AT5G13320 | PBS3 | GAGGGAGAGGAGAAGGAGAC | CGTCTTTGAATCGACATCTCTTG |
| AT5G13930 | TT4 | GGAGATAAAGCTAGGACTAAAGGA | CTAGTATGAAGAGAACGCACGC |
| AT5G24770 | VSP2 | GCCAAAGGACTTGCCCTAAAG | GGTCGGTCTTCTCTGTTCCG |
| AT5G26920 | CBP60G | GGTACGCACCAAGCCGTTG | CCTTAACCTTACACCACCTGG |
| AT5G27420 | CNI1 | GTTGTTCCGTAAAACTCCGTCG | GTCTGCTCGTACCGAGTCAC |
| AT5G39580 | AT5G39580 | CATTTGTACCACAACTTCAAAGAC | CCTCTGTTACGGCTGAGATTG |
| AT5G45110 | NPR3 | GACTTCTTACTCGTATGGTGGC | GAAATCGTCGAGGATGTCGTC |
| AT5G54610 | ANK | CTGCATACGAGAACAATGACAAG | GTTCATGTGAGATCCTCTGGC |
| AT5G56970 | CKX3 | CATGGCTTAATCTCTTCGTACC | CAAGAACAGGACCGCTAGTG |
| AT5G59220 | HAI | CGTGTTATCTACTGGGATGGC | GACCGTCACTTGCGAGAATAAG |
| AT5G60390 | EF1 | GGTGACGCTGGTATGGTTAAG | GTCTGCCTCATGTCCCTAAC |
| AT5G61890 | AT5G61890 | CTCTACATATTACTCCTCCAACC | GCATATCACCACTACTCGCATC |
| AT5G64770 | RGF9 | CTCATACTCTTCATTTCCTCTCC | CGTGCCTTATTTTCCGACTCTC |
