# Supplementary material for: Arabidopsis Transcriptome Analysis Reveals Key Roles of Melatonin in Plant Defense Systems
Source: PLoS One. 2014 Mar 28;9(3):e93462. doi: 10.1371/journal.pone.0093462 (PMC3969325; doi:10.1371/journal.pone.0093462)
Supplement: Table S2 — List of genes that are significantly affected by 100 pM melatonin. (DOCX) [file pone.0093462.s004.docx]

**Table S2:** List of genes that are significantly (p<0.05) affected by 100 pM melatonin.

| Accession # |
| --- |
| AT1G30530 |
| AT4G16590 |
| AT3G45590 |
| AT5G63190 |
| AT5G23670 |
| AT1G28330 |
| AT5G66400 |
| AT4G17470 |
| AT1G70185 |
| AT4G01080 |
| AT2G37900 |
| AT2G44140 |
| AT5G24780 |
| AT5G24770 |
| AT4G16240 |
| AT5G27490 |
| AT3G28220 |
| AT2G46570 |
| AT1G54040 |
| AT3G14730 |
| AT3G45140 |
| AT5G51174 |
| AT4G15440 |
| AT2G34430 |
| AT3G47340 |
| AT4G23600 |
| AT4G08870 |
| AT2G05070 |
| AT4G28040 |
| AT2G39850 |
| AT1G67800 |
| AT5G53420 |
| AT1G02850 |
| AT1G67810 |
| AT3G26440 |
| AT3G44740 |
| AT3G47750 |
| AT3G54826 |
| AT1G15510 |
| AT3G12220 |
| AT3G01420 |
| AT1G77760 |
| AT1G26250 |
| AT3G17050 |
| AT4G39366 |
| AT1G05120 |
| AT2G30750 |
| AT4G24000 |
| AT4G38920 |
| AT3G46220 |
| AT3G23120 |
| AT1G45688 |
| AT3G50480 |
| CUFF.1921 |
| AT4G15390 |
| AT3G11340 |
| AT1G49570 |
| AT3G23450 |
| AT1G26240 |
| AT4G33720 |
| AT3G03670 |
| AT3G47347 |
| AT3G57260 |
| AT1G55310 |
| AT1G17170 |
| AT1G07400 |
| AT5G54610 |
| AT3G05950 |
| AT3G25250 |
| AT5G52520 |
| AT2G14610 |
| AT1G32360 |
| AT2G05755 |
| CUFF.5105 |
| AT3G01180 |
| AT3G20380 |
| AT1G05680 |
| AT1G01440 |
| AT3G05545 |
| AT1G16110 |
| AT1G17720 |
