# Supplementary material for: Arabidopsis Transcriptome Analysis Reveals Key Roles of Melatonin in Plant Defense Systems
Source: PLoS One. 2014 Mar 28;9(3):e93462. doi: 10.1371/journal.pone.0093462 (PMC3969325; doi:10.1371/journal.pone.0093462)
Supplement: Table S3 — List of genes that are significantly affected by 1 mM melatonin. (DOCX) [file pone.0093462.s005.docx]

**Table S3:** List of genes that are significantly (p<0.05) affected by 1 mM melatonin.

| Accession # |
| --- |
| AT1G70530 |
| AT1G70760 |
| AT2G28760 |
| AT2G29890 |
| AT2G43560 |
| AT3G15310 |
| AT4G19530 |
| AT5G45670 |
| AT5G63190 |
| AT1G01010 |
| AT1G01390 |
| AT1G01560 |
| AT1G01680 |
| AT1G01720 |
| AT1G01790 |
| AT1G02205 |
| AT1G02390 |
| AT1G02420 |
| AT1G02450 |
| AT1G02580 |
| AT1G02850 |
| AT1G02900 |
| AT1G02930 |
| AT1G03130 |
| AT1G03220 |
| AT1G03290 |
| AT1G03310 |
| AT1G03600 |
| AT1G03630 |
| AT1G03820 |
| AT1G04040 |
| AT1G04240 |
| AT1G04250 |
| AT1G04520 |
| AT1G04580 |
| AT1G04680 |
| AT1G04980 |
| AT1G05100 |
| AT1G05120 |
| AT1G05260 |
| AT1G05320 |
| AT1G05560 |
| AT1G05690 |
| AT1G05850 |
| AT1G06350 |
| AT1G06680 |
| AT1G07000 |
| AT1G07040 |
| AT1G07320 |
| AT1G07440 |
| AT1G07810 |
| AT1G08090 |
| AT1G08115 |
| AT1G08380 |
| AT1G08630 |
| AT1G09080 |
| AT1G09380 |
| AT1G09500 |
| AT1G09530 |
| AT1G09645 |
| AT1G09750 |
| AT1G10070 |
| AT1G10140 |
| AT1G10150 |
| AT1G10270 |
| AT1G10340 |
| AT1G10360 |
| AT1G10640 |
| AT1G10657 |
| AT1G10760 |
| AT1G11080 |
| AT1G11545 |
| AT1G11700 |
| AT1G11720 |
| AT1G11840 |
| AT1G11850 |
| AT1G12290 |
| AT1G12900 |
| AT1G13340 |
| AT1G13480 |
| AT1G13650 |
| AT1G14020 |
| AT1G14150 |
| AT1G14270 |
| AT1G14345 |
| AT1G14350 |
| AT1G14540 |
| AT1G14550 |
| AT1G14600 |
| AT1G14880 |
| AT1G15010 |
| AT1G15030 |
| AT1G15290 |
| AT1G15380 |
| AT1G15405 |
| AT1G15410 |
| AT1G15510 |
| AT1G15780 |
| AT1G15810 |
| AT1G15980 |
| AT1G16110 |
| AT1G16150 |
| AT1G16390 |
| AT1G16400 |
| AT1G16410 |
| AT1G17500 |
| AT1G17650 |
| AT1G17830 |
| AT1G17860 |
| AT1G18200 |
| AT1G18250 |
| AT1G18300 |
| AT1G18610 |
| AT1G18730 |
| AT1G19020 |
| AT1G19150 |
| AT1G19220 |
| AT1G19250 |
| AT1G19330 |
| AT1G19350 |
| AT1G19510 |
| AT1G19530 |
| AT1G19670 |
| AT1G20010 |
| AT1G20160 |
| AT1G20190 |
| AT1G20220 |
| AT1G21240 |
| AT1G21250 |
| AT1G21390 |
| AT1G21430 |
| AT1G21500 |
| AT1G21525 |
| AT1G21550 |
| AT1G21670 |
| AT1G21750 |
| AT1G22065 |
| AT1G22160 |
| AT1G22400 |
| AT1G22630 |
| AT1G23080 |
| AT1G23205 |
| AT1G23550 |
| AT1G24020 |
| AT1G24070 |
| AT1G24100 |
| AT1G24140 |
| AT1G26130 |
| AT1G26230 |
| AT1G26240 |
| AT1G26250 |
| AT1G26390 |
| AT1G26440 |
| AT1G26761 |
| AT1G26945 |
| AT1G27730 |
| AT1G28260 |
| AT1G28290 |
| AT1G29170 |
| AT1G29290 |
| AT1G29430 |
| AT1G29490 |
| AT1G29500 |
| AT1G29520 |
| AT1G29640 |
| AT1G29670 |
| AT1G29720 |
| AT1G29910 |
| AT1G29920 |
| AT1G29930 |
| AT1G29980 |
| AT1G30220 |
| AT1G30520 |
| AT1G30530 |
| AT1G30620 |
| AT1G30700 |
| AT1G30730 |
| AT1G30760 |
| AT1G31710 |
| AT1G32170 |
| AT1G32360 |
| AT1G32470 |
| AT1G32900 |
| AT1G32960 |
| AT1G33960 |
| AT1G35140 |
| AT1G35230 |
| AT1G35420 |
| AT1G35560 |
| AT1G35710 |
| AT1G43800 |
| AT1G43900 |
| AT1G44000 |
| AT1G44130 |
| AT1G44446 |
| AT1G44575 |
| AT1G45145 |
| AT1G45191 |
| AT1G45201 |
| AT1G47480 |
| AT1G47720 |
| AT1G47860 |
| AT1G48100 |
| AT1G48260 |
| AT1G48600 |
| AT1G49050 |
| AT1G49200 |
| AT1G49570 |
| AT1G49720 |
| AT1G49860 |
| AT1G49975 |
| AT1G50730 |
| AT1G50740 |
| AT1G50750 |
| AT1G51790 |
| AT1G51830 |
| AT1G52030 |
| AT1G52190 |
| AT1G52220 |
| AT1G52230 |
| AT1G52710 |
| AT1G52890 |
| AT1G53040 |
| AT1G53070 |
| AT1G53440 |
| AT1G53510 |
| AT1G53520 |
| AT1G53625 |
| AT1G53700 |
| AT1G53800 |
| AT1G54010 |
| AT1G54020 |
| AT1G54040 |
| AT1G54100 |
| AT1G54730 |
| AT1G54780 |
| AT1G55370 |
| AT1G55780 |
| AT1G56660 |
| AT1G57630 |
| AT1G58520 |
| AT1G59590 |
| AT1G59660 |
| AT1G60060 |
| AT1G60390 |
| AT1G60590 |
| AT1G60800 |
| AT1G61140 |
| AT1G61255 |
| AT1G61520 |
| AT1G61600 |
| AT1G61800 |
| AT1G61820 |
| AT1G62300 |
| AT1G62330 |
| AT1G62540 |
| AT1G62790 |
| AT1G62870 |
| AT1G63040 |
| AT1G63420 |
| AT1G63530 |
| AT1G63720 |
| AT1G63840 |
| AT1G64150 |
| AT1G64160 |
| AT1G64170 |
| AT1G64390 |
| AT1G64680 |
| AT1G64770 |
| AT1G65040 |
| AT1G65230 |
| AT1G65480 |
| AT1G65481 |
| AT1G65610 |
| AT1G65690 |
| AT1G65730 |
| AT1G65860 |
| AT1G65900 |
| AT1G65960 |
| AT1G66090 |
| AT1G66130 |
| AT1G66280 |
| AT1G66400 |
| AT1G66570 |
| AT1G66760 |
| AT1G66830 |
| AT1G66880 |
| AT1G67070 |
| AT1G67350 |
| AT1G67590 |
| AT1G67700 |
| AT1G67750 |
| AT1G67800 |
| AT1G67810 |
| AT1G67900 |
| AT1G68290 |
| AT1G68410 |
| AT1G68620 |
| AT1G68690 |
| AT1G68890 |
| AT1G68940 |
| AT1G69530 |
| AT1G69610 |
| AT1G69840 |
| AT1G69880 |
| AT1G69930 |
| AT1G70140 |
| AT1G70210 |
| AT1G70280 |
| AT1G70370 |
| AT1G70410 |
| AT1G70420 |
| AT1G70820 |
| AT1G70830 |
| AT1G70890 |
| AT1G71500 |
| AT1G71720 |
| AT1G71910 |
| AT1G71960 |
| AT1G72030 |
| AT1G72120 |
| AT1G72130 |
| AT1G72500 |
| AT1G72560 |
| AT1G72645 |
| AT1G72680 |
| AT1G72810 |
| AT1G72930 |
| AT1G72970 |
| AT1G73100 |
| AT1G73310 |
| AT1G73330 |
| AT1G73480 |
| AT1G73640 |
| AT1G73805 |
| AT1G73810 |
| AT1G73885 |
| AT1G74010 |
| AT1G74020 |
| AT1G74070 |
| AT1G74360 |
| AT1G74670 |
| AT1G74710 |
| AT1G75000 |
| AT1G75040 |
| AT1G75240 |
| AT1G75690 |
| AT1G76470 |
| AT1G76520 |
| AT1G76570 |
| AT1G76590 |
| AT1G76600 |
| AT1G76650 |
| AT1G76790 |
| AT1G76880 |
| AT1G76900 |
| AT1G76970 |
| AT1G76980 |
| AT1G77490 |
| AT1G77510 |
| AT1G77530 |
| AT1G78020 |
| AT1G78060 |
| AT1G78265 |
| AT1G78420 |
| AT1G78600 |
| AT1G78770 |
| AT1G78960 |
| AT1G78995 |
| AT1G79075 |
| AT1G79450 |
| AT1G79900 |
| AT1G80130 |
| AT1G80160 |
| AT1G80440 |
| AT1G80450 |
| AT1G80640 |
| AT1G80690 |
| AT2G01180 |
| AT2G01590 |
| AT2G01760 |
| AT2G02020 |
| AT2G02230 |
| AT2G02380 |
| AT2G02710 |
| AT2G02990 |
| AT2G03550 |
| AT2G03850 |
| AT2G04270 |
| AT2G04380 |
| AT2G04430 |
| AT2G04790 |
| AT2G05070 |
| AT2G05210 |
| AT2G05380 |
| AT2G05755 |
| AT2G05940 |
| AT2G07677 |
| AT2G07722 |
| AT2G07827 |
| AT2G10940 |
| AT2G11910 |
| AT2G12461 |
| AT2G13550 |
| AT2G13790 |
| AT2G14560 |
| AT2G14620 |
| AT2G15020 |
| AT2G15050 |
| AT2G15310 |
| AT2G15490 |
| AT2G15690 |
| AT2G16720 |
| AT2G16900 |
| AT2G17500 |
| AT2G17710 |
| AT2G17880 |
| AT2G17972 |
| AT2G18030 |
| AT2G18220 |
| AT2G18300 |
| AT2G18328 |
| AT2G18660 |
| AT2G18690 |
| AT2G18700 |
| AT2G19580 |
| AT2G19590 |
| AT2G20142 |
| AT2G20670 |
| AT2G21050 |
| AT2G21330 |
| AT2G21370 |
| AT2G22170 |
| AT2G22230 |
| AT2G22470 |
| AT2G22990 |
| AT2G23110 |
| AT2G23150 |
| AT2G23170 |
| AT2G23450 |
| AT2G23600 |
| AT2G23672 |
| AT2G23810 |
| AT2G24160 |
| AT2G24270 |
| AT2G25090 |
| AT2G25460 |
| AT2G26020 |
| AT2G26215 |
| AT2G26400 |
| AT2G27150 |
| AT2G27330 |
| AT2G27390 |
| AT2G27402 |
| AT2G27500 |
| AT2G27550 |
| AT2G27720 |
| AT2G28400 |
| AT2G28460 |
| AT2G28550 |
| AT2G28620 |
| AT2G28950 |
| AT2G29420 |
| AT2G29460 |
| AT2G29470 |
| AT2G29480 |
| AT2G29490 |
| AT2G29630 |
| AT2G29940 |
| AT2G30210 |
| AT2G30695 |
| AT2G31020 |
| AT2G31790 |
| AT2G31865 |
| AT2G31880 |
| AT2G31945 |
| AT2G32140 |
| AT2G32160 |
| AT2G32250 |
| AT2G32290 |
| AT2G32500 |
| AT2G32640 |
| AT2G32660 |
| AT2G32700 |
| AT2G32720 |
| AT2G32990 |
| AT2G33330 |
| AT2G33380 |
| AT2G33580 |
| AT2G34410 |
| AT2G34430 |
| AT2G34500 |
| AT2G34680 |
| AT2G35130 |
| AT2G35290 |
| AT2G35710 |
| AT2G35760 |
| AT2G35860 |
| AT2G35960 |
| AT2G35980 |
| AT2G36120 |
| AT2G36220 |
| AT2G36770 |
| AT2G36780 |
| AT2G36800 |
| AT2G36870 |
| AT2G37040 |
| AT2G37450 |
| AT2G37530 |
| AT2G37640 |
| AT2G37750 |
| AT2G37760 |
| AT2G37770 |
| AT2G38120 |
| AT2G38210 |
| AT2G38240 |
| AT2G38250 |
| AT2G38330 |
| AT2G38540 |
| AT2G38640 |
| AT2G39030 |
| AT2G39250 |
| AT2G39340 |
| AT2G39470 |
| AT2G39518 |
| AT2G39530 |
| AT2G39700 |
| AT2G39730 |
| AT2G39850 |
| AT2G40000 |
| AT2G40100 |
| AT2G40150 |
| AT2G40205 |
| AT2G40610 |
| AT2G40750 |
| AT2G41080 |
| AT2G41230 |
| AT2G41380 |
| AT2G41820 |
| AT2G42220 |
| AT2G42380 |
| AT2G42890 |
| AT2G43100 |
| AT2G43310 |
| AT2G43570 |
| AT2G43820 |
| AT2G43900 |
| AT2G44130 |
| AT2G44290 |
| AT2G44920 |
| AT2G44940 |
| AT2G45220 |
| AT2G45470 |
| AT2G45760 |
| AT2G45910 |
| AT2G46535 |
| AT2G46650 |
| AT2G46750 |
| AT2G46820 |
| AT2G46830 |
| AT2G46890 |
| AT2G47130 |
| AT2G47170 |
| AT2G47200 |
| AT2G47240 |
| AT2G47590 |
| AT2G47720 |
| AT2G47860 |
| AT2G47950 |
| AT3G01290 |
| AT3G01440 |
| AT3G01490 |
| AT3G01500 |
| AT3G01550 |
| AT3G01660 |
| AT3G01810 |
| AT3G02020 |
| AT3G02380 |
| AT3G02875 |
| AT3G03190 |
| AT3G03310 |
| AT3G03560 |
| AT3G03640 |
| AT3G03780 |
| AT3G04000 |
| AT3G04070 |
| AT3G04140 |
| AT3G04290 |
| AT3G04300 |
| AT3G04320 |
| AT3G04630 |
| AT3G05220 |
| AT3G05545 |
| AT3G05600 |
| AT3G05730 |
| AT3G05900 |
| AT3G05950 |
| AT3G06035 |
| AT3G06145 |
| AT3G06500 |
| AT3G06700 |
| AT3G06850 |
| AT3G06880 |
| AT3G06895 |
| AT3G06900 |
| AT3G08520 |
| AT3G08940 |
| AT3G09010 |
| AT3G09070 |
| AT3G09270 |
| AT3G09650 |
| AT3G10040 |
| AT3G10060 |
| AT3G10160 |
| AT3G10300 |
| AT3G10340 |
| AT3G10520 |
| AT3G10815 |
| AT3G11000 |
| AT3G11120 |
| AT3G11340 |
| AT3G11480 |
| AT3G11820 |
| AT3G12220 |
| AT3G12610 |
| AT3G12830 |
| AT3G13180 |
| AT3G13240 |
| AT3G13310 |
| AT3G13380 |
| AT3G13520 |
| AT3G13610 |
| AT3G13670 |
| AT3G14420 |
| AT3G14620 |
| AT3G14870 |
| AT3G15030 |
| AT3G15550 |
| AT3G15720 |
| AT3G15850 |
| AT3G16130 |
| AT3G16240 |
| AT3G16340 |
| AT3G16370 |
| AT3G17050 |
| AT3G17180 |
| AT3G18000 |
| AT3G18050 |
| AT3G18320 |
| AT3G18890 |
| AT3G19000 |
| AT3G19370 |
| AT3G19450 |
| AT3G19480 |
| AT3G19550 |
| AT3G19580 |
| AT3G19710 |
| AT3G19850 |
| AT3G20380 |
| AT3G20430 |
| AT3G20470 |
| AT3G20490 |
| AT3G20760 |
| AT3G21040 |
| AT3G21460 |
| AT3G21700 |
| AT3G21760 |
| AT3G21770 |
| AT3G22060 |
| AT3G22142 |
| AT3G22200 |
| AT3G22231 |
| AT3G22234 |
| AT3G22550 |
| AT3G22600 |
| AT3G22910 |
| AT3G22930 |
| AT3G23070 |
| AT3G23090 |
| AT3G23450 |
| AT3G23810 |
| AT3G23830 |
| AT3G23890 |
| AT3G24090 |
| AT3G24500 |
| AT3G25250 |
| AT3G25290 |
| AT3G25610 |
| AT3G25882 |
| AT3G25980 |
| AT3G26060 |
| AT3G26180 |
| AT3G26210 |
| AT3G26230 |
| AT3G26280 |
| AT3G26440 |
| AT3G26470 |
| AT3G26510 |
| AT3G26520 |
| AT3G26670 |
| AT3G26910 |
| AT3G27170 |
| AT3G27400 |
| AT3G28080 |
| AT3G28210 |
| AT3G28220 |
| AT3G28270 |
| AT3G28300 |
| AT3G28340 |
| AT3G28510 |
| AT3G28540 |
| AT3G29030 |
| AT3G29320 |
| AT3G32980 |
| AT3G33000 |
| AT3G33002 |
| AT3G42660 |
| AT3G43790 |
| AT3G44990 |
| AT3G45860 |
| AT3G45970 |
| AT3G46080 |
| AT3G46090 |
| AT3G46220 |
| AT3G46250 |
| AT3G46660 |
| AT3G46690 |
| AT3G46780 |
| AT3G46970 |
| AT3G47340 |
| AT3G47430 |
| AT3G47470 |
| AT3G47480 |
| AT3G47750 |
| AT3G47800 |
| AT3G48360 |
| AT3G48420 |
| AT3G48460 |
| AT3G48580 |
| AT3G48650 |
| AT3G48720 |
| AT3G49120 |
| AT3G49130 |
| AT3G49290 |
| AT3G49780 |
| AT3G50260 |
| AT3G50470 |
| AT3G50480 |
| AT3G50740 |
| AT3G50750 |
| AT3G50770 |
| AT3G51150 |
| AT3G51510 |
| AT3G51660 |
| AT3G51860 |
| AT3G52150 |
| AT3G52370 |
| AT3G52400 |
| AT3G52750 |
| AT3G53190 |
| AT3G53260 |
| AT3G53800 |
| AT3G54040 |
| AT3G54050 |
| AT3G54600 |
| AT3G54750 |
| AT3G54826 |
| AT3G54890 |
| AT3G54960 |
| AT3G55080 |
| AT3G55120 |
| AT3G55340 |
| AT3G55430 |
| AT3G55470 |
| AT3G55630 |
| AT3G55880 |
| AT3G55970 |
| AT3G56030 |
| AT3G56160 |
| AT3G56400 |
| AT3G56710 |
| AT3G57260 |
| AT3G58120 |
| AT3G58820 |
| AT3G59000 |
| AT3G59060 |
| AT3G59100 |
| AT3G59300 |
| AT3G59350 |
| AT3G59770 |
| AT3G59930 |
| AT3G59940 |
| AT3G60130 |
| AT3G60140 |
| AT3G60420 |
| AT3G60540 |
| AT3G60966 |
| AT3G61070 |
| AT3G61390 |
| AT3G61490 |
| AT3G61870 |
| AT3G62550 |
| AT3G62750 |
| AT3G63160 |
| AT3G63200 |
| AT3G63370 |
| AT3G63380 |
| AT4G00020 |
| AT4G00165 |
| AT4G00360 |
| AT4G00490 |
| AT4G00670 |
| AT4G00880 |
| AT4G01250 |
| AT4G01360 |
| AT4G01380 |
| AT4G01430 |
| AT4G01870 |
| AT4G01935 |
| AT4G01950 |
| AT4G01995 |
| AT4G02280 |
| AT4G02290 |
| AT4G02380 |
| AT4G02550 |
| AT4G03060 |
| AT4G03298 |
| AT4G03400 |
| AT4G03450 |
| AT4G04490 |
| AT4G04500 |
| AT4G04620 |
| AT4G04692 |
| AT4G04750 |
| AT4G04830 |
| AT4G07507 |
| AT4G08555 |
| AT4G08685 |
| AT4G08770 |
| AT4G08780 |
| AT4G08950 |
| AT4G09000 |
| AT4G10060 |
| AT4G10090 |
| AT4G10340 |
| AT4G10380 |
| AT4G10390 |
| AT4G10860 |
| AT4G10970 |
| AT4G11170 |
| AT4G11211 |
| AT4G11290 |
| AT4G11320 |
| AT4G11460 |
| AT4G11650 |
| AT4G11890 |
| AT4G12030 |
| AT4G12310 |
| AT4G12320 |
| AT4G12390 |
| AT4G12400 |
| AT4G12490 |
| AT4G12545 |
| AT4G12580 |
| AT4G12720 |
| AT4G12800 |
| AT4G12830 |
| AT4G12880 |
| AT4G12917 |
| AT4G13390 |
| AT4G13395 |
| AT4G13420 |
| AT4G13495 |
| AT4G13505 |
| AT4G13510 |
| AT4G13555 |
| AT4G13770 |
| AT4G13810 |
| AT4G13840 |
| AT4G14100 |
| AT4G14130 |
| AT4G14368 |
| AT4G14370 |
| AT4G14630 |
| AT4G15390 |
| AT4G15396 |
| AT4G15540 |
| AT4G16240 |
| AT4G16380 |
| AT4G16660 |
| AT4G16730 |
| AT4G16740 |
| AT4G17770 |
| AT4G17810 |
| AT4G18010 |
| AT4G18050 |
| AT4G18950 |
| AT4G18970 |
| AT4G19170 |
| AT4G19380 |
| AT4G19520 |
| AT4G19880 |
| AT4G20430 |
| AT4G20760 |
| AT4G20830 |
| AT4G20860 |
| AT4G20940 |
| AT4G21180 |
| AT4G21300 |
| AT4G21380 |
| AT4G21580 |
| AT4G21585 |
| AT4G21670 |
| AT4G21870 |
| AT4G22070 |
| AT4G22200 |
| AT4G22290 |
| AT4G22470 |
| AT4G22520 |
| AT4G22540 |
| AT4G22570 |
| AT4G22610 |
| AT4G22890 |
| AT4G23190 |
| AT4G23460 |
| AT4G23470 |
| AT4G23680 |
| AT4G23690 |
| AT4G24000 |
| AT4G24040 |
| AT4G24160 |
| AT4G24700 |
| AT4G24780 |
| AT4G24810 |
| AT4G25000 |
| AT4G25080 |
| AT4G25100 |
| AT4G25310 |
| AT4G25434 |
| AT4G25600 |
| AT4G25810 |
| AT4G26060 |
| AT4G26070 |
| AT4G26200 |
| AT4G26230 |
| AT4G26470 |
| AT4G26530 |
| AT4G26950 |
| AT4G27030 |
| AT4G27370 |
| AT4G27410 |
| AT4G27440 |
| AT4G28040 |
| AT4G28250 |
| AT4G28490 |
| AT4G29030 |
| AT4G29510 |
| AT4G29700 |
| AT4G30020 |
| AT4G30170 |
| AT4G30280 |
| AT4G30650 |
| AT4G30710 |
| AT4G31240 |
| AT4G31850 |
| AT4G32260 |
| AT4G32450 |
| AT4G32800 |
| AT4G32950 |
| AT4G33070 |
| AT4G33150 |
| AT4G33220 |
| AT4G33270 |
| AT4G33420 |
| AT4G33540 |
| AT4G33720 |
| AT4G33910 |
| AT4G34050 |
| AT4G34120 |
| AT4G34135 |
| AT4G34138 |
| AT4G34220 |
| AT4G34380 |
| AT4G34600 |
| AT4G34710 |
| AT4G36040 |
| AT4G36360 |
| AT4G36540 |
| AT4G36640 |
| AT4G36850 |
| AT4G36988 |
| AT4G37010 |
| AT4G37370 |
| AT4G37770 |
| AT4G37800 |
| AT4G37925 |
| AT4G37990 |
| AT4G38660 |
| AT4G38770 |
| AT4G38840 |
| AT4G38860 |
| AT4G38950 |
| AT4G39210 |
| AT4G39330 |
| AT4G39363 |
| AT4G39510 |
| AT4G39670 |
| AT4G39710 |
| AT4G39830 |
| AT4G39960 |
| AT5G01015 |
| AT5G01100 |
| AT5G01380 |
| AT5G02160 |
| AT5G02890 |
| AT5G02930 |
| AT5G03190 |
| AT5G03350 |
| AT5G03760 |
| AT5G04190 |
| AT5G04310 |
| AT5G04360 |
| AT5G04930 |
| AT5G05190 |
| AT5G05250 |
| AT5G05270 |
| AT5G05365 |
| AT5G05410 |
| AT5G05580 |
| AT5G05790 |
| AT5G05960 |
| AT5G06310 |
| AT5G06860 |
| AT5G07010 |
| AT5G07030 |
| AT5G07100 |
| AT5G07440 |
| AT5G07460 |
| AT5G08000 |
| AT5G08720 |
| AT5G09220 |
| AT5G09580 |
| AT5G09820 |
| AT5G10170 |
| AT5G10460 |
| AT5G10580 |
| AT5G10625 |
| AT5G10695 |
| AT5G11210 |
| AT5G11420 |
| AT5G11520 |
| AT5G11790 |
| AT5G11920 |
| AT5G13080 |
| AT5G13190 |
| AT5G13200 |
| AT5G13220 |
| AT5G13320 |
| AT5G13330 |
| AT5G13770 |
| AT5G13930 |
| AT5G14200 |
| AT5G14420 |
| AT5G14470 |
| AT5G14610 |
| AT5G14660 |
| AT5G14930 |
| AT5G15410 |
| AT5G15790 |
| AT5G15840 |
| AT5G16030 |
| AT5G16170 |
| AT5G16300 |
| AT5G16360 |
| AT5G16530 |
| AT5G16715 |
| AT5G16910 |
| AT5G16970 |
| AT5G17330 |
| AT5G17520 |
| AT5G17670 |
| AT5G17760 |
| AT5G18260 |
| AT5G18270 |
| AT5G18460 |
| AT5G18570 |
| AT5G18780 |
| AT5G19090 |
| AT5G19730 |
| AT5G19750 |
| AT5G20230 |
| AT5G20240 |
| AT5G20410 |
| AT5G20540 |
| AT5G20935 |
| AT5G21100 |
| AT5G21430 |
| AT5G22210 |
| AT5G22300 |
| AT5G22340 |
| AT5G22580 |
| AT5G23020 |
| AT5G23060 |
| AT5G23100 |
| AT5G23210 |
| AT5G23670 |
| AT5G23830 |
| AT5G23870 |
| AT5G24030 |
| AT5G24105 |
| AT5G24200 |
| AT5G24205 |
| AT5G24210 |
| AT5G24530 |
| AT5G25190 |
| AT5G25250 |
| AT5G25440 |
| AT5G25610 |
| AT5G25770 |
| AT5G25920 |
| AT5G25930 |
| AT5G26170 |
| AT5G26220 |
| AT5G26280 |
| AT5G26690 |
| AT5G26770 |
| AT5G26920 |
| AT5G27240 |
| AT5G27280 |
| AT5G27300 |
| AT5G27420 |
| AT5G27490 |
| AT5G27660 |
| AT5G27730 |
| AT5G28020 |
| AT5G28450 |
| AT5G33355 |
| AT5G35480 |
| AT5G35490 |
| AT5G35580 |
| AT5G36170 |
| AT5G36220 |
| AT5G36790 |
| AT5G37020 |
| AT5G37790 |
| AT5G38140 |
| AT5G38430 |
| AT5G38510 |
| AT5G38710 |
| AT5G39050 |
| AT5G39210 |
| AT5G39510 |
| AT5G39580 |
| AT5G39610 |
| AT5G39670 |
| AT5G39970 |
| AT5G40010 |
| AT5G40380 |
| AT5G40390 |
| AT5G40730 |
| AT5G41050 |
| AT5G41140 |
| AT5G41460 |
| AT5G41610 |
| AT5G41750 |
| AT5G42070 |
| AT5G42100 |
| AT5G42770 |
| AT5G42800 |
| AT5G43340 |
| AT5G43370 |
| AT5G43380 |
| AT5G43580 |
| AT5G43750 |
| AT5G43870 |
| AT5G43910 |
| AT5G43980 |
| AT5G43990 |
| AT5G44410 |
| AT5G44420 |
| AT5G44600 |
| AT5G44680 |
| AT5G44990 |
| AT5G45000 |
| AT5G45110 |
| AT5G45630 |
| AT5G45650 |
| AT5G45680 |
| AT5G45820 |
| AT5G45930 |
| AT5G45950 |
| AT5G46110 |
| AT5G46230 |
| AT5G46730 |
| AT5G47330 |
| AT5G47500 |
| AT5G48180 |
| AT5G48380 |
| AT5G48400 |
| AT5G48540 |
| AT5G48545 |
| AT5G48657 |
| AT5G48870 |
| AT5G48900 |
| AT5G49450 |
| AT5G49690 |
| AT5G49730 |
| AT5G50335 |
| AT5G50740 |
| AT5G51460 |
| AT5G51480 |
| AT5G51630 |
| AT5G51680 |
| AT5G51720 |
| AT5G52050 |
| AT5G52100 |
| AT5G52390 |
| AT5G52750 |
| AT5G52760 |
| AT5G52800 |
| AT5G52860 |
| AT5G52970 |
| AT5G53090 |
| AT5G53120 |
| AT5G53420 |
| AT5G53870 |
| AT5G53880 |
| AT5G54040 |
| AT5G54075 |
| AT5G54270 |
| AT5G54570 |
| AT5G54610 |
| AT5G54650 |
| AT5G54770 |
| AT5G54860 |
| AT5G55620 |
| AT5G55730 |
| AT5G55740 |
| AT5G55970 |
| AT5G56540 |
| AT5G56840 |
| AT5G56850 |
| AT5G56970 |
| AT5G57340 |
| AT5G57480 |
| AT5G57510 |
| AT5G57550 |
| AT5G57560 |
| AT5G57655 |
| AT5G57770 |
| AT5G58005 |
| AT5G58260 |
| AT5G58300 |
| AT5G59020 |
| AT5G59220 |
| AT5G59390 |
| AT5G59690 |
| AT5G59780 |
| AT5G59820 |
| AT5G59870 |
| AT5G60450 |
| AT5G60890 |
| AT5G60930 |
| AT5G61010 |
| AT5G61030 |
| AT5G61390 |
| AT5G61560 |
| AT5G61820 |
| AT5G61890 |
| AT5G62020 |
| AT5G62140 |
| AT5G62280 |
| AT5G62350 |
| AT5G62360 |
| AT5G62480 |
| AT5G62630 |
| AT5G63180 |
| AT5G63530 |
| AT5G63680 |
| AT5G63700 |
| AT5G63760 |
| AT5G63790 |
| AT5G63970 |
| AT5G63990 |
| AT5G64190 |
| AT5G64240 |
| AT5G64260 |
| AT5G64380 |
| AT5G64460 |
| AT5G64770 |
| AT5G64810 |
| AT5G64860 |
| AT5G65020 |
| AT5G65210 |
| AT5G65310 |
| AT5G65960 |
| AT5G66310 |
| AT5G66400 |
| AT5G67080 |
| AT5G67280 |
| AT5G67340 |
| AT5G67360 |
| AT5G67385 |
| AT5G67390 |
| AT5G67600 |
| CUFF.1189 |
| CUFF.1191 |
| CUFF.5105 |
