# Supplementary material for: Arabidopsis Transcriptome Analysis Reveals Key Roles of Melatonin in Plant Defense Systems
Source: PLoS One. 2014 Mar 28;9(3):e93462. doi: 10.1371/journal.pone.0093462 (PMC3969325; doi:10.1371/journal.pone.0093462)
Supplement: Table S4 — qRT-PCR validation of RNA-seq data. (DOCX) [file pone.0093462.s006.docx]

| Table S4: qRT-PCR validation of RNA-seq data of 60 selected genes. qRT-PCR was conducted 4 times for each gene. * p<0.05, ** p<0.01, ***p<0.001. | | | | | | | | | | |
| --- | --- | --- | --- | --- | --- | --- | --- | --- | --- | --- |
|  |  | **100 pM Melatonin** | | | | **1 mM Melatonin** | | | | |
|  |  | mRNA-seq | | qPCR | | mRNA-seq | | qPCR | | |
| Accession # | Gene name | fold change | | 2^-∆∆ct | | fold change | | 2^-∆∆ct | | |
| AT1G01310 | CAP | -2.84 | *** | -3.49 | *** | -5.72 | *** | | -10.91 | *** |
| AT4G15390 | AT4G15390 | -2.40 | ** | -1.73 | ** | -4.73 | *** | | -2.12 | * |
| AT1G74670 | GASA6 | 0.51 |  | 2.01 |  | -4.33 | *** | | -3.07 | ** |
| AT5G61890 | AT5G61890 | -0.28 |  | 2.19 |  | -4.24 | *** | | 1.19 |  |
| AT5G13930 | TT4 | 0.04 |  | 1.09 |  | -3.87 | *** | | -3.84 | *** |
| AT1G04250 | AXR3 | -0.53 |  | 1.20 |  | -3.49 | *** | | -1.97 | ** |
| AT3G28220 | AT3G28220 | 2.95 | *** | 3.55 | ** | -3.42 | *** | | -1.48 |  |
| AT4G13770 | CYP83A1 | -0.14 |  | 1.01 |  | -3.39 | *** | | -5.53 | *** |
| AT2G26020 | PDF | 0.21 |  | 1.92 |  | -3.29 | *** | | -1.10 |  |
| AT3G57260 | BGL2 | -1.11 |  | -1.10 |  | -3.05 | *** | | 1.56 |  |
| AT5G64770 | RGF9 | 0.22 |  | 1.59 |  | -3.01 | *** | | -3.99 | * |
| AT3G04290 | LTL1 | -0.34 |  | 1.09 |  | -2.81 | *** | | -9.08 | *** |
| AT4G25100 | FSD1 | 0.00 |  | -1.05 |  | -2.80 | *** | | -6.65 | ** |
| AT1G28290 | AGP31 | -0.57 |  | 1.13 |  | -2.58 | *** | | -3.03 | ** |
| AT3G20470 | GRP5 | 0.95 |  | 1.34 | * | -2.35 | *** | | -1.43 | * |
| AT2G38210 | PDX1L4 | 0.48 |  | 1.80 |  | -2.02 | *** | | -1.95 | ** |
| AT1G19670 | ATCLH1 | 1.07 |  | 1.96 | ** | -2.01 | *** | | -2.30 | *** |
| AT4G23810 | WRKY53 | -0.64 |  | 2.52 | * | -0.94 |  | | 1.26 | * |
| AT5G24770 | VSP2 | 2.48 | *** | 7.16 | *** | 0.76 |  | | 2.01 | * |
| AT4G39030 | EDS5 | -0.90 |  | 1.03 | *** | 1.20 | * | | 2.23 | *** |
| AT2G14610 | PR1 | -4.15 | *** | -1.65 |  | 1.68 | * | | 2.56 | * |
| AT5G07010 | ST2A | 0.13 |  | -1.32 |  | 2.01 | *** | | 4.89 | ** |
| AT2G27150 | AAO3 | 0.10 |  | 1.25 | * | 2.01 | *** | | 4.93 | ** |
| AT5G45110 | NPR3 | -0.25 |  | 1.35 | * | 2.05 | *** | | 5.21 | *** |
| AT5G13320 | PBS3 | -0.68 |  | -1.01 |  | 2.05 | *** | | 18.34 | *** |
| AT2G40000 | HSPRO2 | 0.94 |  | 2.38 | ** | 2.06 | *** | | 4.78 | *** |
| AT1G74020 | SS2 | -0.56 |  | -1.01 |  | 2.09 | *** | | 4.49 | ** |
| AT1G05560 | UGT75B1 | 0.00 |  | 2.00 |  | 2.09 | *** | | 6.34 | *** |
| AT3G52400 | SYP122 | -0.03 |  | 1.34 | * | 2.09 | *** | | 3.45 | *** |
| AT4G08950 | EXO | 0.09 |  | 1.70 |  | 2.12 | *** | | 5.30 | ** |
| AT3G28210 | PMZ | -2.04 | ** | -1.45 |  | 2.12 | *** | | 6.92 | ** |
| AT1G54100 | ALDH7B4 | 0.24 |  | 2.15 |  | 2.16 | *** | | 5.22 | *** |
| AT4G34138 | UGT73B1 | 0.39 |  | 1.29 |  | 2.16 | *** | | 6.81 | *** |
| AT5G13190 | GILP | -0.29 | * | 1.46 | ** | 2.20 | *** | | 6.10 | ** |
| AT2G23170 | GH3.3 | -1.30 |  | 1.97 |  | 2.23 | *** | | 3.65 | * |
| AT2G29420 | ATGSTU7 | -0.50 |  | -1.18 |  | 2.23 | *** | | 9.73 | ** |
| AT2G36800 | DOGT1 | -1.00 |  | 1.21 |  | 2.25 | *** | | 7.33 | *** |
| AT2G33380 | RD20 | 0.60 |  | 1.57 | * | 2.28 | *** | | 4.33 | ** |
| AT3G49780 | ATPSK4 | 0.02 |  | 3.57 | * | 2.32 | *** | | 11.81 | * |
| AT1G73805 | SARD1 | -0.36 |  | 1.25 |  | 2.41 | *** | | 4.43 | *** |
| AT3G22231 | PCC1 | -1.57 | ** | -1.73 | * | 2.47 | *** | | 7.34 | *** |
| AT5G27420 | CNI1 | -0.40 |  | 2.09 |  | 2.48 | *** | | 7.30 | *** |
| AT3G55970 | JRG21 | -0.32 |  | -1.06 |  | 2.49 | *** | | 7.29 | * |
| AT2G41230 | ORS1 | -1.09 |  | 1.00 |  | 2.61 | *** | | 8.15 | *** |
| AT2G32140 | AT2G32140 | -0.47 |  | 1.51 | * | 2.63 | *** | | 3.09 | * |
| AT3G22060 | AT3G22060 | -0.63 |  | 1.84 |  | 2.64 | *** | | 6.19 | * |
| AT1G63840 | AT1G63840 | -0.46 |  | 1.11 |  | 2.77 | *** | | 6.61 | *** |
| AT1G54040 | ESP | 2.87 |  | 1.21 |  | 2.94 | *** | | 1.27 |  |
| AT1G76650 | CML38 | 1.77 | * | 1.30 |  | 2.95 | *** | | 4.02 | ** |
| AT5G59220 | HAI1 | 0.02 |  | 1.42 | * | 3.01 | *** | | 4.57 | *** |
| AT2G29490 | ATGSTU1 | -1.56 |  | 1.45 | * | 3.10 | *** | | 2.51 | ** |
| AT4G26200 | ACS7 | 0.66 |  | 1.74 | * | 3.43 | *** | | 7.02 | *** |
| AT5G26920 | CBP60G | 0.08 |  | 1.25 | * | 3.52 | *** | | 9.74 | *** |
| AT5G39580 | AT5G39580 | 0.62 |  | 1.50 | * | 3.63 | *** | | 14.66 | *** |
| AT5G54610 | ANK | -1.42 | * | -1.59 | ** | 3.72 | *** | | 17.30 | * |
| AT2G25090 | CIPK16 | 0.02 |  | 1.45 |  | 3.83 | *** | | 1.90 | ** |
| AT1G02450 | NIMIN1 | -1.13 |  | -1.14 |  | 4.02 | *** | | 5.17 | *** |
| AT4G16740 | ATTPS03 | -1.74 |  | 1.01 |  | 4.10 | *** | | 1.65 |  |
| AT5G56970 | CKX3 | -0.55 |  | 1.50 |  | 4.52 | *** | | 1.71 |  |
| AT3G11480 | BSMT1 | 0.61 |  | 1.15 |  | 5.79 | *** | | 1.52 | * |
