# Supplementary material for: Arabidopsis Transcriptome Analysis Reveals Key Roles of Melatonin in Plant Defense Systems
Source: PLoS One. 2014 Mar 28;9(3):e93462. doi: 10.1371/journal.pone.0093462 (PMC3969325; doi:10.1371/journal.pone.0093462)
Supplement: Table S5 — Genes with changes in expression levels of at least 2 fold in response to 1 mM Melatonin involved in one or more hormone signaling pathways. (DOCX) [file pone.0093462.s007.docx]

| **Table S5:** Genes with changes in expression levels of at least 2 fold in response to 1 mM Melatonin involved in one or more hormone signaling pathways. | | | | | | | | |  |
| --- | --- | --- | --- | --- | --- | --- | --- | --- | --- |
| **Accession #** | **Gene** | **Description** | **Fold Change** | **Hormone Involved** | | | | | |
|  |  |  |  | Auxin | ABA | SA | JA | Ethylene | |
| **AT5G63190** | MDC12 | MA3 domain-containing protein | 1.79769e+308 | **+** |  |  |  |  | |
| **AT3G11480** | BSMT1 | SABATH methyltransferase | 5.79226 |  |  | **+** | **+** |  | |
| **AT5G13320** | PBS3 | AVRPPHB SUSCEPTIBLE 3 | 5.21030 | **+** | **+** | **+** | **+** |  | |
| **AT3G28510** | AT3G28510 | P-loop containing nucleoside triphosphate hydrolase | 4.25263 |  |  | **+** |  |  | |
| **AT2G26400** | ARD3 | Acireductone dioxygenase 3 | 4.23087 |  |  | **+** |  |  | |
| **AT4G16740** | TPS03 | (E,E)-alpha-farnesene synthase | 4.09933 |  |  |  | **+** |  | |
| **AT1G02450** | NIMIN1 | NIM1-interacting 1 | 4.01997 |  |  | **+** | **+** |  | |
| **AT5G64810** | WRKY51 | WRKY DNA-binding protein 51 | 3.89444 |  |  |  | **+** |  | |
| **AT2G14560** | LURP1 | Late upregulated in response to Hyaloperonospora parasitica | 3.81235 |  |  | **+** | **+** |  | |
| **AT1G33960** | AIG1 | AVRRPT2-induced gene 1 | 3.79304 |  |  | **+** |  |  | |
| **AT5G54610** | ANK | Ankyrin repeat family protein | 3.71571 |  |  | **+** | **+** |  | |
| **AT4G03450** | AT4G03450 | Ankyrin repeat family protein | 3.70179 |  |  | **+** |  |  | |
| **AT5G26170** | WRKY50 | WRKY DNA-binding protein 51 | 3.60762 |  |  |  | **+** |  | |
| **AT2G35980** | YLS9 | Yellow-leaf-specific gene 9 | 3.56127 |  |  | **+** | **+** |  | |
| **AT1G75040** | PR5 | Pathogenesis-related gene 5 | 3.54955 |  |  | **+** | **+** |  | |
| **AT5G26920** | CBP60G | Calmodulin binding protein 60-like G | 3.51898 |  |  | **+** | **+** |  | |
| **AT4G26200** | ACS7 | 1-Amino-cyclopropane-1-carboxylate synthase 7 | 3.42945 |  |  |  |  | **+** | |
| **AT4G39670** | AT4G39670 | Glycolipid transfer protein | 3.40463 |  | **+** | **+** | **+** | **+** | |
| **AT1G74710** | EDS16 | Enhanced disease susceptibility to erysiphe orontii 16 | 3.39868 |  |  | **+** | **+** |  | |
| **AT1G19250** | FMO1 | Flavin-dependent monooxygenase 1 | 3.35557 |  |  | **+** |  |  | |
| **AT1G66090** | AT1G66090 | Disease resistance protein (TIR-NBS class) | 3.16883 |  |  | **+** | **+** |  | |
| **AT2G45760** | BAP2 | BON association protein 2 | 3.15121 |  |  | **+** |  | **+** | |
| **AT5G52050** | AT5G52050 | MATE efflux family protein | 3.12576 |  |  |  |  | **+** | |
| **AT2G29450** | ATGSTU1 | TAU glutathione S-transferase family protein | 3.09797 | **+** |  |  |  |  | |
| **AT1G66760** | AT1G66760 | MATE efflux family protein | 3.08580 |  |  |  | **+** |  | |
| **AT1G01720** | NAC002 | NAC domain containing protein 2 | 3.07132 |  | **+** |  | **+** |  | |
| **AT2G31945** | AT2G31945 | unknown protein | 3.06727 |  |  |  |  | **+** | |
| **AT4G01870** | AT4G01870 | TolB related protein | 3.05516 |  | **+** |  |  | **+** | |
| **AT5G59220** | HAI1 | highly ABA-induced PP2C gene 1 | 3.00759 | **+** | **+** | **+** | **+** | **+** | |
| **AT1G76650** | CML38 | Calmodulin-like 38 | 2.94656 |  | **+** |  | **+** | **+** | |
| **AT1G54040** | ESP | Epithiospecifier protein | 2.94039 |  |  |  | **+** |  | |
| **AT1G19020** | AT1G19020 | unknown function | 2.92043 |  |  | **+** |  |  | |
| **AT5G66400** | ATDI8 | Arabidopsis thaliana drought-induced 8 | 2.88202 |  | **+** |  |  |  | |
| **AT5G44990** | AT5G44990 | Glutathione S-transferase family protein | 2.84051 |  |  |  |  | **+** | |
| **AT1G63840** | AT1G63840 | RING/U-box superfamily protein | 2.77456 | **+** | **+** | **+** | **+** | **+** | |
| **AT3G56400** | WRKY70 | WRKY DNA-binding protein 70 | 2.74608 |  |  | **+** | **+** |  | |
| **AT4G37770** | ACS8 | Encodes an auxin inducible ACC synthase. | 2.73046 | **+** |  |  |  | **+** | |
| **AT5G13080** | WRKY75 | WRKY DNA-binding protein 75 | 2.71688 |  |  |  |  | **+** | |
| **AT1G63720** | AT1G63720 | Hydroxyproline-rich glycoprotein family protein | 2.70803 | **+** | **+** | **+** | **+** | **+** | |
| **AT5G57560** | TCH4 | Xyloglucan endotransglucosylase/hydrolase 22 | 2.67655 | **+** |  |  |  |  | |
| **AT3G22060** | AT3G22060 | Cysteine-rich repeat secretory protein 38 | 2.64093 |  | **+** |  |  |  | |
| **AT3G48360** | BT2 | BTB and TAZ domain protein 2 | 2.63736 | **+** | **+** | **+** | **+** |  | |
| **AT1G57630** | AT1G57630 | Toll-Interleukin-Resistance domain family protein | 2.62980 |  |  | **+** | **+** |  | |
| **AT2G32140** | AT2G32140 | Transmembrane receptor protein | 2.62540 |  |  |  |  | **+** | |
| **AT5G40010** | AATP1 | ATPase-in-Seed-Development | 2.59647 |  |  | **+** |  |  | |
| **AT3G51660** | AT3G51660 | Tautomerase/MIF superfamily protein | 2.58788 | **+** |  |  |  |  | |
| **AT3G50260** | CEJ1 | Cooperatively regulated by ethylene and jasmonate 1 | 2.57229 |  | **+** | **+** |  |  | |
| **AT1G65690** | AT1G65690 | Late embryogenesis abundant hydroxyproline-rich glycoprotein family | 2.56997 |  |  | **+** |  |  | |
| **AT1G21390** | emb2170 | embryo defective 2170 | 2.55431 |  |  |  |  | **+** | |
| **AT2G15490** | UGT73B4 | UDP-glycosyltransferase 73B4 | 2.54687 |  |  |  |  | **+** | |
| **AT5G25930** | AT5G25930 | Leucine-rich repeat receptor-like protein kinase | 2.53548 |  | **+** |  |  | **+** | |
| **AT1G24140** | AT1G24140 | putative metalloproteinase | 2.52615 |  |  |  |  | **+** | |
| **AT4G11890** | ARCK1 | ABA- and osmotic-stress-inducible receptor-like cytosolic kinase 1 | 2.50129 |  | **+** | **+** | **+** |  | |
| **AT4G04500** | CRK37 | cysteine-rich receptor-like protein kinase 37 | 2.49604 |  |  | **+** |  |  | |
| **AT3G55970** | JRG21 | Jasmonate-regulated gene 21 | 2.48874 |  |  |  | **+** |  | |
| **AT5G27420** | CNI1 | Carbon/Nitrogen Insensitive 1; a RING type ubiquitin ligase | 2.48246 | **+** | **+** | **+** | **+** | **+** | |
| **AT3G56710** | SIB1 | Sig1 binding protein | 2.46591 |  |  | **+** |  |  | |
| **AT5G20230** | BCB | Blue copper binding protein | 2.46029 |  |  |  | **+** |  | |
| **AT1G22400** | ATUGT85A1 | Arabidopsis thaliana UDP-glucosyl transferase 85A1 | 2.45489 |  |  | **+** | **+** |  | |
| **AT5G67080** | MAPKKK19 | Mitogen-activated protein kinase kinase kinase 19 | 2.43614 |  | **+** |  |  | **+** | |
| **AT3G47480** | AT3G47480 | Calcium-binding EF-hand family protein | 2.42378 |  |  | **+** | **+** |  | |
| **AT1G04980** | ATPDI10 | Arabidopsis thaliana protein disulfide isomerase 10 | 2.41684 |  |  | **+** |  |  | |
| **AT2G05940** | RIPK | RPM1-induced protein kinase | 2.41642 |  | **+** | **+** | **+** |  | |
| **AT1G73805** | SARD1 | SAR Deficient 1 | 2.41244 |  |  | **+** | **+** |  | |
| **AT5G59820** | RHL41 | Responsive to high light 41 | 2.39584 |  |  |  | **+** |  | |
| **AT5G06860** | ATPGIP1 | Polygalacturonase inhibiting protein 1 | 2.37120 |  | **+** | **+** | **+** |  | |
| **AT5G61820** | AT5G61820 | unknown protein | 2.35544 |  | **+** |  |  |  | |
| **AT2G17500** | MJB20.6 | Auxin efflux carrier family protein | 2.35123 | **+** |  |  |  |  | |
| **AT5G38710** | AT5G38710 | Methylenetetrahydrofolate reductase family protein | 2.33313 |  |  | **+** | **+** |  | |
| **AT5G52760** | AT5G52760 | Copper transport protein family | 2.33313 |  |  | **+** | **+** |  | |
| **AT1G13340** | AT1G13340 | Regulator of Vps4 activity in the MVB pathway protein | 2.30883 |  |  | **+** |  |  | |
| **AT1G10340** | AT1G10340 | Ankyrin repeat family protein | 2.30758 |  |  | **+** |  |  | |
| **AT1G01560** | MPK11 | MAP Kinase 11 | 2.30442 | **+** | **+** | **+** | **+** | **+** | |
| **AT2G33380** | RD20 | Responsive to dessication 20 | 2.28141 |  | **+** | **+** |  |  | |
| **AT2G16720** | MYB7 | MYB domain protein 7 | 2.26732 |  | **+** | **+** | **+** | **+** | |
| **AT1G76970** | AT1G76970 | Target of Myb protein 1 | 2.26000 |  |  | **+** |  |  | |
| **AT5G63970** | RGLG3 | Ring domain ligase 3 | 2.25577 |  |  |  | **+** |  | |
| **AT5G67340** | AT5G67340 | ARM repeat superfamily protein | 2.23994 |  | **+** | **+** | **+** |  | |
| **AT2G29420** | GSTU7 | Glutathione S-transferase tau 7 | 2.22849 |  |  | **+** |  |  | |
| **AT2G23170** | GH3.3 | Indole-3-acetic acid-amido synthetase | 2.22620 | **+** |  |  |  |  | |
| **AT3G19580** | ZF2 | Zinc-finger protein 2 | 2.20418 | **+** | **+** | **+** | **+** | **+** | |
| **AT3G60420** | AT3G60420 | Phosphoglycerate mutase family protein | 2.20370 |  |  | **+** |  |  | |
| **AT3G60540** | AT3G60540 | Sec61-beta subunit protein | 2.20180 |  |  | **+** |  |  | |
| **AT5G11520** | ASP3 | Chloroplastic aspartate aminotransferase | 2.19792 |  |  |  |  | **+** | |
| **AT5G13190** | GILP | GSH-induced LITAF domain protein | 2.19572 |  |  | **+** | **+** |  | |
| **AT1G52890** | NAC019 | NAC domain containing protein 19 | 2.19063 | **+** | **+** | **+** | **+** | **+** | |
| **AT3G12830** | SAUR72 | Small Auxin upregulated 72 | 2.17823 | **+** |  |  |  |  | |
| **AT4G34138** | UGT73B1 | UDP-glucosyl transferase 73B1 | 2.16144 |  | **+** |  |  |  | |
| **AT1G54100** | ALDH7B4 | Aldehyde dehydrogenase | 2.15863 | **+** | **+** | **+** |  | **+** | |
| **AT3G28340** | GATL10 | Galacturonosyltransferase-like 10 | 2.15611 |  | **+** |  |  |  | |
| **AT1G70530** | CPK3 | cysteine-rich receptor-like protein kinase 3 | 2.14698 |  |  | **+** |  |  | |
| **AT1G72680** | ATCAD1 | Cinnamyl alcohol dehydrogenase 1 | 2.12583 |  |  | **+** |  |  | |
| **AT3G28210** | PMZ | Zinc finger protein | 2.11933 | **+** | **+** | **+** | **+** | **+** | |
| **AT4G04490** | CRK36 | cysteine-rich receptor-like protein kinase | 2.11580 |  | **+** | **+** | **+** |  | |
| **AT2G43820** | UGT74F2 | UDP-Glucosyltransferase 74F2 | 2.11424 |  |  | **+** |  |  | |
| **AT4G26470** | AT4G26470 | Calmodulin-like protein 21 | 2.10667 |  |  |  |  | **+** | |
| **AT1G05560** | UGT75B1 | UDP-glucose transferase 75B1 | 2.08708 |  | **+** | **+** |  |  | |
| **AT1G73480** | AT1G73480 | Alpha/beta-Hydrolases superfamily protein | 2.08676 |  | **+** |  |  |  | |
| **AT1G74020** | SS2 | Strictosidine synthase 2 | 2.08580 |  |  |  | **+** |  | |
| **AT2G05380** | GRP3S | glycine-rich protein 3 short isoform | 2.08059 | **+** |  |  |  |  | |
| **AT1G76600** | AT1G76600 | unknown protein | 2.07886 |  |  |  |  | **+** | |
| **AT3G52400** | SYP122 | syntaxin protein | 2.06173 | **+** | **+** | **+** | **+** | **+** | |
| **AT1G21250** | WAK1 | Cell wall-associated kinase 1 | 2.05986 |  |  | **+** | **+** |  | |
| **AT2G40000** | HSPRO2 | Ortholog of sugar beet HS1 PRO-1 2 | 2.05724 |  |  | **+** |  |  | |
| **AT1G21750** | PDIL1-1 | Protein disulfide isomerase-like protein | 2.05650 |  |  | **+** |  |  | |
| **AT1G27730** | STZ | Salt-tolerance zinc finger | 2.05565 | **+** | **+** | **+** | **+** | **+** | |
| **AT2G40750** | WRKY54 | WRKY DNA-binding protein 54 | 2.05047 |  |  | **+** | **+** |  | |
| **AT5G45110** | NPR3 | NPR1-like protein 3 | 2.04961 |  |  | **+** | **+** |  | |
| **AT3G24500** | MBF1C | Multiprotein bridging factor 1C | 2.04727 |  | **+** |  |  | **+** | |
| **AT1G77510** | PDIL1-2 | Protein disulfide isomerase-like 1-2 | 2.04159 |  |  | **+** |  |  | |
| **AT2G18690** | AT2G18690 | unknown function | 2.02843 | **+** |  | **+** |  |  | |
| **AT5G24530** | DMR6 | Downy mildew resistance 6 protein | 2.01769 |  |  | **+** | **+** |  | |
| **AT2G27150** | AAO3 | Abscisic aldehyde oxidase 3 | 2.01291 |  | **+** |  |  |  | |
| **AT3G26210** | CYP71B23 | Cytochrome P450 71B23 | 2.01045 |  |  | **+** |  |  | |
| **AT5G07010** | ST2A | Sulfotransferase 2A | 2.01007 |  |  |  | **+** |  | |
| **AT1G19670** | CLH1 | Chlorophyllase 1 | -2.00664 |  |  | **+** | **+** |  | |
| **AT5G25610** | RD22 | Responsive to dessication 22 | -2.01531 |  | **+** |  |  |  | |
| **AT2G38210** | PDX1L4 | Putative PDX1-like protein 4 | -2.01617 |  |  |  |  | **+** | |
| **AT2G37040** | PAL1 | Phenylalanine ammonia-lyase 1 | -2.04568 |  |  | **+** |  |  | |
| **AT2G46820** | PSI-P | Photosystme I P subunit | -2.05208 |  | **+** | **+** |  |  | |
| **AT2G41820** | AT2G41820 | Leucine-rich repeat protein kinase family protein | -2.06899 | **+** |  |  |  |  | |
| **AT1G70370** | PG2 | Polygalacturonase 2 | -2.11182 | **+** |  |  |  |  | |
| **AT5G48870** | SAD1 | Supersensitive to ABA and Drought 1 | -2.19225 |  | **+** |  |  |  | |
| **AT4G22200** | KT2/3 | potassium transport 2/3 | -2.20297 |  | **+** |  |  |  | |
| **AT4G08770** | Prx37 | Peroxidase 37 | -2.21812 | **+** |  |  |  |  | |
| **AT1G06680** | PSBP-1 | Photosystem II subunit P-1 | -2.25405 |  |  | **+** | **+** |  | |
| **AT2G45470** | FLA8 | FASCICLIN-like arabinogalactan protein 8 | -2.26278 | **+** |  |  |  |  | |
| **AT1G24100** | UGT74B1 | UDP-glucosyl transferase 74B1 | -2.28945 | **+** |  |  |  |  | |
| **AT3G20470** | GRP5 | Glycine-rich protein 5 | -2.34513 |  | **+** | **+** |  |  | |
| **AT1G31710** | AT1G31710 | Copper amine oxidase family protein | -2.37346 | **+** |  |  |  |  | |
| **AT1G10270** | GRP23 | Glycine-rich protein 23 | -2.39835 |  | **+** | **+** |  |  | |
| **AT3G10040** | AT3G10040 | Sequence-specific DNA binding transcription factor | -2.41108 |  |  | **+** |  |  | |
| **AT4G38840** | AT4G38840 | SAUR-like auxin-responsive protein family | -2.42102 | **+** |  |  |  |  | |
| **AT1G05850** | ELP | Endo chitinase-like protein | -2.43396 | **+** | **+** | **+** |  |  | |
| **AT1G53700** | WAG1 | Auxin-induced protein kinase | -2.48533 | **+** |  |  |  |  | |
| **AT2G39730** | RCA | Rubisco activase | -2.50343 |  |  | **+** | **+** |  | |
| **AT1G75240** | HB33 | Homeobox protein 33 | -2.51608 |  | **+** |  |  |  | |
| **AT5G55620** | AT5G55620 | unknown protein | -2.54084 |  |  |  |  | **+** | |
| **AT1G28290** | AGP31 | Arabinogalactan protein 31 | -2.57954 |  |  |  | **+** |  | |
| **AT4G32260** | AT4G32260 | ATP synthase beta chain (Subunit II) | -2.60897 |  |  | **+** | **+** |  | |
| **AT4G38860** | AT4G38860 | SAUR-like auxin-responsive protein family | -2.64102 | **+** |  |  |  |  | |
| **AT1G04680** | AT1G04680 | Pectin lyase-like superfamily protein | -2.68802 | **+** |  |  |  |  | |
| **AT5G48900** | AT5G48900 | Pectin lyase-like superfamily protein | -2.69330 | **+** |  |  |  |  | |
| **AT2G21050** | LAX2 | Like auxin resistant 2 | -2.70401 | **+** |  |  |  |  | |
| **AT2G22170** | PLAT2 | PLAT domain protein 2 | -2.73413 |  |  |  | **+** |  | |
| **AT4G20940** | GHR1 | Guard cell hydrogen peroxide-resistant 1 | -2.76233 |  | **+** |  |  |  | |
| **AT3G04290** | LTL1 | Li-tolerant lipase 1 | -2.80958 |  |  | **+** |  |  | |
| **AT3G16370** | AT3G16370 | GDSL-like Lipase/Acylhydrolase superfamily protein | -2.83692 | **+** |  |  |  |  | |
| **AT5G40390** | SIP1 | Seed imbibition 1-like | -2.86143 |  | **+** |  |  |  | |
| **AT3G55120** | TT5 | Transparent testa 5 | -2.86673 | **+** | **+** | **+** |  |  | |
| **AT5G60890** | MYB34 | Myb-like transcription factor 34 | -2.96740 |  |  |  | **+** |  | |
| **AT5G64770** | RGF9 | Root meristem growth factor 9 | -3.00840 | **+** |  |  |  |  | |
| **AT2G21330** | FBA1 | Fructose-bisphosphate aldolase 1 | -3.03127 |  |  |  | **+** |  | |
| **AT3G57260** | BGL2 | Beta-1,3-glucanase 2 | -3.04964 |  |  | **+** | **+** |  | |
| **AT4G12030** | BAT5 | Bile acid transporter 5 | -3.07104 |  |  |  | **+** |  | |
| **AT3G01500** | CA1 | Beta-carbonic anhydrase 1 | -3.08196 |  |  | **+** | **+** |  | |
| **AT4G33070** | PDC1 | Pyruvate decarboxylase 1 | -3.08596 |  |  | **+** |  |  | |
| **AT4G10390** | AT4G10390 | Receptor-like protein kinase | -3.10278 |  |  |  | **+** |  | |
| **AT2G19590** | ACO1 | ACC oxidase 1 | -3.13848 |  |  |  |  | **+** | |
| **AT2G26020** | PDF1.2b | Putative plant defensin 1.2b | -3.28502 |  |  |  |  | **+** | |
| **AT4G13770** | CYP83A1 | Cytochrome p450 83A1 | -3.38940 | **+** |  |  |  |  | |
| **AT5G23020** | IMS2 | Methyl thioalkymalate synthase-like | -3.46527 | **+** |  | **+** |  |  | |
| **AT1G04250** | AXR3 | Auxin resistant 3 | -3.49394 | **+** |  | **+** |  |  | |
| **AT5G25190** | ESE3 | Ethylene-responsive transcription factor ERF003 | -3.49854 |  |  |  |  | **+** | |
| **AT1G29490** | SAUR68 | Small auxin upregulated 69 | -3.52745 | **+** |  |  |  |  | |
| **AT2G43100** | IPMI2 | Isopropylmalate isomerase 2 | -3.59639 | **+** |  |  |  |  | |
| **AT1G04240** | SHY2 | Short hypocotyl 2 | -3.70592 | **+** |  | **+** |  |  | |
| **AT1G29500** | AT1G29500 | SAUR-like auxin-responsive protein family | -3.84556 | **+** |  |  |  |  | |
| **AT5G45820** | CIPK20 | CBL-interacting protein kinase 20 | -3.86907 |  | **+** |  |  |  | |
| **AT5G13930** | TT4 | Transparent testa 4 | -3.87023 | **+** |  |  | **+** |  | |
| **AT4G13510** | ATAMT1 | Ammonium transporter 1 | -3.89447 |  | **+** | **+** |  |  | |
| **AT1G43800** | FTM1 | Floral transition at the meristem1 | -3.98758 |  |  | **+** |  |  | |
| **AT4G00880** | AT4G00880 | SAUR-like auxin-responsive protein family | -4.09193 | **+** |  |  |  |  | |
| **AT1G49860** | GSTF14 | Glutathione S-transferase (class phi) 14 | -4.10306 |  |  | **+** |  |  | |
| **AT5G44420** | PDF1.2 | Plant defensin 1.2 | -4.10721 |  |  |  | **+** | **+** | |
| **AT5G61890** | ERF114 | Ethylene-responsive transcription factor 114 | -4.23551 |  |  |  |  | **+** | |
| **AT1G29430** | AT1G29431 | SAUR-like auxin-responsive protein family | -4.27318 | **+** |  |  |  |  | |
| **AT2G38120** | AUX1 | Auxin resistant 1 | -4.28187 | **+** |  |  |  | **+** | |
| **AT5G16530** | PIN5 | Auxin efflux carrier component 8 | -4.37990 | **+** |  |  |  |  | |
| **AT3G19710** | BCAT4 | Branched-chain aminotransferase 4 | -4.49660 | **+** |  |  |  |  | |
| **AT1G05690** | BT3 | BTB and TAZ domain protein 3 | -4.59053 |  |  | **+** |  | **+** | |
| **AT5G23670** | LCB2 | Long chain base biosynthesis protein 2 | suppressed |  |  |  |  | **+** | |
