# Supplementary material for: Arabidopsis Transcriptome Analysis Reveals Key Roles of Melatonin in Plant Defense Systems
Source: PLoS One. 2014 Mar 28;9(3):e93462. doi: 10.1371/journal.pone.0093462 (PMC3969325; doi:10.1371/journal.pone.0093462)
Supplement: Table S6 — Downstream genes in plant stress defense that are affected by melatonin and their fold changes. (DOCX) [file pone.0093462.s008.docx]

| **Table S6**. Downstream genes in plant stress defense that are affected by melatonin and their fold changes. | | | |
| --- | --- | --- | --- |
| Gene Name | Description |  | Fold Change |
| AT4G33720 | CAP superfamily protein | | -5.72455 |
| AT3G28220 | TRAF-like family protein | | -3.42054 |
| PDF1.2b | Predicted to encode a PR (pathogenesis-related) protein | | -3.28502 |
| BGL2 | Beta-1,3-Glucanase 2 | | -3.04964 |
| AT1G64160 | Disease resistance-responsive family protein | | -3.02321 |
| ATLP-1 | Thaumatin-like protein | | -2.9891 |
| AT4G23690 | Disease resistance-responsive family protein | | -2.84556 |
| ATOCT3 | Organic cation/carnitine transporter 3 | | -2.52502 |
| ELP | Endo chitinase-like protein AtCTL1 | | -2.43396 |
| NHL12 | Encodes a protein similar to tobacco hairpin-induced gene | | -2.43053 |
| MGD2 | Type B monogalactosyldiacylglycerol (MGDG) synthase | | -2.39476 |
| AT3G62550 | Adenine nucleotide alpha hydrolases-like superfamily protein | | -2.35871 |
| AtMS2 | Cytosolic methionine synthase | | -2.22483 |
| ATPDIL1-2 | Disulfide isomerase-like (PDIL) protein | | 2.04159 |
| RNS1 | Member of the ribonuclease T2 family | | 2.04382 |
| ATPDIL1-1 | Encodes a protein disulfide isomerase-like (PDIL) protein | | 2.0565 |
| AT4G23680 | Polyketide cyclase/dehydrase and lipid transport superfamily protein | | 2.13175 |
| GATL10 | Protein with putative galacturonosyltransferase activity | | 2.15611 |
| AT1G03220 | Eukaryotic aspartyl protease family protein | | 2.16219 |
| AT1G14880 | Plant Cadmium Resistance 1 (PCR1) | | 2.3419 |
| AT2G47130 | Encodes a short-chain dehydrogenase/reductase | | 2.4506 |
| ATBCB | Blue Copper Binding Protein | | 2.46029 |
| PCC1 | A member of a novel 6 member Arabidopsis gene family | | 2.47348 |
| AT3G51660 | Tautomerase/MIF superfamily protein | | 2.58788 |
| ESP | Epithiospecifier protein | | 2.94039 |
| FMO1 | Required for full expression of TIR-NB-LRR–conditioned resistance | | 3.35557 |
| YLS9 | Protein similar to tobacco hairpin-induced gene (HIN1) | | 3.56127 |
| AT2G37770 | Encodes an NADPH-dependent aldo-keto reductase | | 3.66767 |
| ATGSTU3 | Glutathione transferase belonging to the tau class of GSTs | | 3.82212 |
| GLP9 | Germin-like protein | | 4.42415 |
| AT3G59930 | Encodes a defensin-like (DEFL) family protein | | 4.98141 |
| AT5G33355 | Encodes a defensin-like (DEFL) family protein | | 5.00801 |
